# Exploratory multivariate analysis using R Language for method development in liquid chromatography

**DOI:** 10.1007/s00216-024-05705-y

**Published:** 2025-01-10

**Authors:** Miloš Hroch

**Affiliations:** Department of Medical Biochemistry, Faculty of Medicine in Hradec Králové, Charles University, Šimkova 870, 500 03 Hradec Králové, Czech Republic

**Keywords:** R Language, Optimization, Factor analysis of mixed data, Hierarchical clustering, Liquid chromatography

## Abstract

The visual evaluation of data derived from screening and optimization experiments in the development of new analytical methods poses a considerable time investment and introduces the risk of subjectivity. This study presents a novel approach to processing such data, based on factor analysis of mixed data and hierarchical clustering — multivariate techniques implemented in the R programming language. The methodology is demonstrated in the early-stage screening and optimization of the chromatographic separation of 15 structurally diverse drugs that affect the central nervous system, using a custom R Language script. The presented explorative approach enabled the identification of key parameters affecting the separation and significantly reduced the time required to evaluate the comprehensive dataset from the screening experiments. Based on the data analysis results, the optimal combination of stationary phase and mobile phase composition was selected, considering retention, overall resolution, and peak shape of compounds. Additionally, compounds vulnerable to changes in selected chromatographic conditions were identified. As a complement to the presented R Language script, a web-based application ChromaFAMDeX has been developed to offer an intuitive interface that enhances the accessibility of the used statistical methods. Accompanying the publication, the R script and the link to the standalone application are provided, enabling replication and adaptation of the methodology.

**Graphical Abstract:**

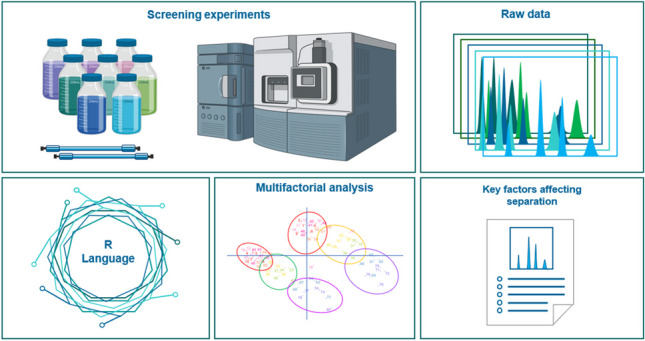

**Supplementary Information:**

The online version contains supplementary material available at 10.1007/s00216-024-05705-y.

## Introduction

Liquid chromatography-mass spectrometry (LC–MS) is an indispensable tool across various disciplines. Despite the emergence of shotgun methodologies that solely rely on the capabilities of the mass spectrometer [1], the role of liquid chromatography remains critical in numerous instances [2]. Implementing separation offers substantial benefits, particularly in scenarios involving the analysis of isobaric compounds [3], addressing matrix effects in complex biological samples [4], co-eluting analytes [5], or situations where separation facilitates preferential use of non-overlapping scan segments in mass spectrometric detection [6], thus improving method robustness and selectivity [7].

However, chromatography optimization can impose a significant burden on personnel and instrument time. Moreover, instances of empirical trial-and-error optimization still persist, possibly leading to suboptimal results and lengthy method development. This reality emphasizes the need for structured strategies, leading to the transition from traditional practices to systematic, data-driven approaches that are focused on the principles of quality-by-design (QbD), thus allowing the identification and a greater understanding of factors that influence the performance of the method [8].

The traditional practice of ad hoc optimization has also been significantly changed by the broader adoption of computer-assisted method development tools. Software applications such as DryLab or ChromSword provide a systematic approach to retention modeling, typically based on initial data derived from exploratory chromatographic runs conducted under predefined conditions [9]. In recent years, open-source platforms like the R Language have gained attention for their flexibility and availability [10]. R Language offers a broad spectrum of packages for data processing, statistical analysis, modeling, and visualization, which are often missing in common spreadsheet calculators or statistical software. In particular, several R-based packages have been developed to address specific tasks in chromatography. These include the RChrom package facilitating computer-assisted retention modeling [11], RpeakChrom supporting the characterization of chromatographic columns [12], and chromatographR, allowing LC-DAD data analysis [13].

Despite these advances, a universal and widely accessible tool for rapid evaluating chromatographic raw data generated during LC method development and optimization remains unavailable. Chemometric tools primarily rely on response surface methodologies or factorial designs [14], while multivariate statistical methods such as principal component analysis (PCA) [15–17], or partial least squares (PLS) [18] are largely focused on interpreting assays’ results, particularly for classification and analysis of complex datasets across various research areas [19]. The application of multivariate statistical tools in method evaluation is scarce. One notable exception is the work of Felinger et al., who utilized PCA to evaluate the reproducibility of retention times across different columns and batches [20]. Nevertheless, the specific applications of multivariate techniques capable of handling mixed data types in LC method optimization remain underexplored.

To address this gap, the primary objective of the presented study was to demonstrate possibilities of multivariate exploratory methods for evaluating chromatographic data. Among the broad array of available statistical tools, factor analysis of mixed data (FAMD) [21, 22] and hierarchical clustering (HC) [23] were selected. The primary reason for implementing FAMD lies in its significant advantage over PCA, as it enables the direct analysis of datasets containing both continuous and categorical variables (mixed data). The applicability of this approach is demonstrated through a specific case study in early-stage method development, where the effects of factors such as the type of stationary phase, organic solvent, pH, and mobile phase modifier on separation of compounds were assessed. For this demonstration, 15 structurally distinct drugs affecting the central nervous system were selected as model compounds. These included antidepressants (citalopram, trazodone, mirtazapine, sertraline), antipsychotics (olanzapine, amisulpride, quetiapine, haloperidol, flupentixol), benzodiazepines (zolpidem, clonazepam, alprazolam, diazepam), and drugs of abuse (methamphetamine, fentanyl). The acquired data were processed using a custom R script. The outputs of the FAMD and HC analyses provided a rapid overview of basic trends in the acquired chromatographic data, enabling the identification of key parameters affecting retention and peak distortion, as well as showing similarities in the results. Furthermore, a web-based application ChromaFAMDeX was developed to facilitate the use of presented methods without requiring knowledge of R language, or direct interaction with the R script, thereby promoting broader adoption of the methodology. It is important to note that the presented methodology can also be implemented in other statistical software and is not restricted to R Language, highlighting its versatility. In conclusion, to the best of my knowledge, this study represents the first implementation of FAMD and HC in LC separation methods evaluation, showing its novel application in the field.

## Material and methods

### Materials

Solutions (1 mg/mL in methanol) of citalopram, trazodone, mirtazapine, sertraline, methamphetamine, fentanyl, olanzapine, amisulpride, quetiapine, haloperidol, flupentixol, zolpidem, clonazepam, alprazolam, and diazepam as certified reference material were purchased from Merck (Prague, Czech Republic). Honeywell LC–MS-grade acetonitrile, methanol, formic acid, acetic acid, and ammonium hydroxide were purchased from Avantor (Prague, Czech Republic). LC–MS-grade ammonium formate and ammonium acetate were purchased from Merck (Prague, Czech Republic). Ultrapure water was dispensed by a Milli-Q system (Merck, Prague, Czech Republic).

### Samples and solutions

The stock solutions of the compounds, as supplied by the manufacturer, were stored at − 80 °C. Testing samples at a concentration of 20 ng/mL in 10% v/v acetonitrile were freshly prepared each day prior to analysis. The testing samples were maintained at 10 °C in an autosampler unit during analysis.

### Liquid chromatography

A liquid chromatography was performed on the Acquity I-Class chromatographic system (Waters, USA) using the stationary phases Triart C18, Triart C18 ExRS, Triart Phenyl (50 × 2.1 mm, 1.9 µm, YMC, Japan), Acquity BEH C18 (50 × 2.1 mm, 1.7 µm, Waters, USA), and Luna OMEGA Polar C18 (50 × 2.1 mm, 1.6 µm, Phenomenex, USA). The columns were protected with an Acquity in-line filter (Waters, USA). The following generic gradient at 40 °C and a flow rate of 0.35 mL/min was used in initial screening experiments: 10–100% solvent B in 0.00–8.0 min, 100% solvent B in 8.0–10.0 min, and 10% solvent B in 10.0–13.0 min for column equilibration. Water was used as solvent A and 95% (v/v) methanol or acetonitrile in water as solvent B; both solvents with additives are given in Table S1 (Suppl. Information).

The final optimized gradient involved water (solvent A) and 95% (v/v) methanol in water (solvent B), both with 5 mM NH_4_HCO_3_ and 0.02% (v/v) ammonia. The temperature was 60 °C, and flow rate was 0.3 mL/min. The gradient profile was as follows: 40–55% solvent B in 0–2.5 min, 55% solvent B in 2.5–3.5 min, 55–65% solvent B in 3.5–4.2 min, 65% solvent B in 4.2–5.5 min, 65–80% solvent B in 5.5–6.5 min, 80% solvent B in 6.5–7.5 min, and final re-equilibration with 40% solvent B in 7.6–10 min. A sample volume of 5 µL was injected for analysis.

### Mass spectrometry

The Xevo TQ-XS mass spectrometer (Waters, USA) was operated with an ESI interface in positive ion mode. The optimized MS parameters are summarized in Table S2 (Suppl. Information). MassLynx software (Waters, USA) was employed for data acquisition.

### Dataset preparation and computation

The raw data from LC–MS were processed using TargetLynx (Waters, USA), exported to Microsoft Excel 365, and saved as a TAB-delimited text file for analysis in R. The exported data contained independent descriptors of the separation method, such as the stationary phase, organic solvent, mobile phase additive, and pH; and a set of dependent parameters included retention times and peak skewness of compounds. These data were completed with a unique ID (Suppl. Information, Table S1) for each combination of independent chromatographic parameters (individual), sample peak capacity (PkC**) as an estimate of overall resolution calculated according to Dolan et al. [24], and mean absolute peak skewness, corresponding to a deviation from ideal peak symmetry, calculated using the following equation:

$$\overline{{P }_{sk}}=\left[ \frac{\sum_{i=1}^{n}{{abs(P}_{sk}}_{i})}{n}\right]$$

where ${{P}_{sk}}_{i}$ is the peak skewness for the *i*th compound and $n$ is the number of compounds in the chromatographic run. An example of resulting dataset layout is presented in Table S6 (Suppl. Information). Statistical analysis and visualization in R Language (version 4.3.2) were performed using RStudio for Windows (Version 2023.12.1), utilizing packages FactoMineR (Version 2.8) [25], Factoextra (Version 1.0.7) [26], Factoshiny (Version 2.4) [27], caret (Version 6.0.94) [28], rgl (Version 1.3.1) [29], ape (Version 5.7.1) [30], rsm (Version 2.10.5) [31], ggrepel (Version 0.9.5) [32], and ggplot2 (Version 3.4.3) [33]. A web-based application ChromaFAMDeX coded using Shiny package [34] is accessible via ShinyApps platform at https://chromafamdex.shinyapps.io/ChromaFAMDeX/.

### Factor analysis of mixed data

The central to the FAMD analysis using FactoMineR library is *FAMD()* function performing all necessary calculations including input data normalization. The results of the analysis performed by *FAMD()* function were captured in the *res.famd* object. In the next step, eigenvalues were extracted using the *get_eigenvalue()* function to assess the amount of variance explained by each principal dimension (equivalent to principal component in PCA), thus guiding the selection of significant principal dimensions for processing. Subsequently, an evaluation of the principal dimensions was conducted, focusing on the first two, which are typically the most informative. This was achieved through the *dimdesc$* function, to identify variables significantly correlated (*p* < 0.05) with these dimensions. Further, information on variables, including their coordinates in the new coordinate system, quality of representation, and their contribution to the explained variability in the dataset, was extracted from the *res.famd* object using *get_famd_var()* function. The individual observations were extracted using *get_famd_ind()* function for a more granular analysis of their positioning and relationships within the new dimensions.

The outputs of the analysis included the following: (a) A scree plot to assess the eigenvalue distribution, providing insights into the data variability explained by each principal dimension and guiding the selection of principal dimensions for further analysis. (b) A correlation cycle to visualize correlations between quantitative variables and the principal dimensions and correlation patterns among quantitative variables themselves. A color gradient indicated the strength of each variable’s contribution to the explained variability. (c) A bar plot of variables’ contribution to the principal dimensions Dim 1 and Dim 2 to highlight the variables significantly contributing to the first two principal dimensions. (d) A factorial map to illustrate the contribution of qualitative variables to Dim 1 and Dim 2, using a color gradient to indicate contribution levels. (e) A plot of individuals, showing the spatial distribution of individuals in Dim 1 and Dim 2, using the *habillage* parameter to classify individuals according to additive in the mobile phase, organic solvent, or stationary phase. Spearman’s correlation analysis was performed to confirm results obtained in the correlation circle. The R language script is provided in Suppl. Information (Sections. 3 and 4).

### Factor analysis of mixed data: K-fold cross-validation

To evaluate the robustness of the FAMD method, K-fold cross-validation, using eight folds (*K* = 8), was employed, and the mean ± SD (*n* = K) was calculated for the resulting new coordinates using R language script (Suppl. Information, Sect. 5).

### Hierarchical clustering

The HC analysis was conducted to explore the similarities and groupings among individuals based on their coordinates derived from FAMD. A distance matrix containing the pairwise straight-line distances between individuals was calculated, using the Euclidean distance method. Subsequently, agglomerative HC was performed, employing Ward’s minimum-variance method, to determine the distances between clusters. The results of the HC analysis were visualized using a circular dendrogram, which was used to facilitate the identification of individuals exhibiting similar qualities. The R Language script is provided in Suppl. Information (Sect. 3.5).

### Box-Behnken design optimization of flow rate and temperature

Optimization of temperature and flow rate was based on a response surface method using Box-Behnken design (BBD) [35]. The BBD experiment was performed at three levels: low (− 1), intermediate (0), and high (1), and results were fitted with second-order polynomial. Column temperature and flow rate were selected as input variables, while the resolution of the critical pair olanzapine-mirtazapine was selected as the response variable. A design matrix listing nine experimental runs (Suppl. Information, Table S7) and resulting surface response plot were generated using R Language script (Suppl. Information, Sect. 6).

### Evaluation of optimal and suboptimal conditions

For the evaluation of retention, the combinations of independent chromatographic parameters that yielded the highest overall retention of compounds were considered optimal. While in the evaluation of peak distortion, the combinations resulting in the smallest overall deviation of peak skewness from zero were deemed optimal, with the opposite conditions considered suboptimal.

## Results and discussion

### Overview

An exploratory multivariate analysis methodology designed for chromatography evaluation was applied on the dataset containing selected descriptors of the separation method. The analysis of such multidimensional structure usually faces challenges from high dimensionality, variables’ interaction, redundancy, and multicollinearity, which obscure the data structure and complicate the isolation of individual variable effects. Conducting a comprehensive visual evaluation of such data is challenging, introduces subjectivity, and fails to reveal hidden dependencies, complicating the rapid identification of critical parameters affecting chromatography.

To process such data, it was first necessary to select an appropriate tool from the R Language portfolio comprising, e.g., correspondence analysis [36], hierarchical clustering (HC) [23], PCA [21], multiple correspondence analysis (MCA) [21], and FAMD [21]. The FAMD was chosen as the most suitable method, combining elements from PCA and MCA. The FAMD offers an advantage over both methods by directly handling mixed data types, enabling the integration of numeric variables and categorical factors into a single dataset. Like PCA and MCA, FAMD is a dimensionality reduction technique that identifies new orthogonal axes (principal dimensions) where the data exhibits the greatest variance. The result is a transformation of the data into a new coordinate system, where the new coordinates are linear combinations of the coordinates representing original variables.

The application of FAMD in LC method development provides valuable insights into the analyzed dataset, as interpreting FAMD outputs can help identify the relative importance of the studied parameters and support targeted optimization of the method under investigation.

1. **Correlation circle analysis**. Inspecting the correlation circle can reveal which numeric variables are correlated and the nature of their correlations. Vectors pointing in the same direction indicate a strong positive correlation, whereas vectors pointing in opposite directions suggest an inverse relationship. Orthogonal vectors indicate uncorrelated variables. The length of a vector in the correlation circle reflects how strongly these variables contribute to the principal dimensions. This output can, for example, reveal the correlation between the retention times of analyzed compounds and pH.
2. **Plot of variable contributions**. The variable contributions plot illustrates how dependent and independent variables contribute to the variability of the original dataset. Variables contributing more to major dimensions have greater impact, while those with minimal influence can be considered less significant. For instance, focusing on independent variables such as the additive in the mobile phase, organic solvent, pH, or stationary phase can identify the parameters that contribute most significantly to the variability of dependent variables, such as retention time, resolution, or peak skewness, thus allowing to identify key parameters affecting chromatographic separation. Conversely, in the case of dependent variables (e.g., retention time or peak skewness), a high contribution of a specific compound(s) to dataset variability indicates that this compound is highly influenced by changes in independent parameters. This highlights the conditions under which changes in the dependent variables are feasible due to alterations in independent parameters and where such relationships do not exist. The most important outcome of analyzing this plot is identifying which independent variables have a significant impact on the observed dependent chromatographic parameters. It also helps determine which compounds are most susceptible to these changes.
3. **Factorial map**. The inspection of the factorial map reveals the spatial distribution of categorical variables within the principal dimensions. The proximity of factors on the factorial map indicates a similar effect on the variability of the dataset, whereas factors located on opposite sides suggest an opposing influence. The position of factors in the new coordinate system provides insight into their contributions to the particular principal component, and color coding further highlights their contributions to dataset variability. By analyzing this plot, it can be determined which combinations of independent chromatographic parameters will have similar or entirely different effects on the chromatographic separation.
4. **Plot of individuals**. The plot of individuals illustrates clustering of individual observations, highlighting similarities and differences among them. This facilitates the identification of individuals that lead to similar chromatographic outcomes. Additionally, the use of color coding (habillage) enables the examination of the influence of categorical variables like stationary phase, additive in mobile phase, or organic solvent on the clustering. In LC method development, this output can assist in selecting alternatives, such as a different stationary phase, or identifying options for achieving an opposing outcome when modifications to the LC method are required. Based on the color coding (habillage) according to examined factors, it can also support conclusions drawn from other outputs of the FAMD analysis. When factorial map and plot of individuals are combined, the resulting plot directly shows how clusters in a plot of individuals are related to a particular factor in factorial map.

In addition to FAMD, the HC was used to organize individuals into groups based on similarity, using all principal dimensions generated by FAMD, producing a dendrogram visually representing the clustering structure of the data. To evaluate the effectiveness of the proposed method, five UHPLC columns and 16 mobile phase compositions were used for the separation of 15 compounds, resulting in a dataset containing 80 unique combinations of independent chromatographic parameters (individuals) assigned with a unique ID (Suppl. Information, Table S1). When applying FAMD to chromatography data evaluation, it was essential to define the specific objectives of the statistical analysis. Here, the primary objective was to identify key parameters influencing chromatographic separation of the evaluated compounds.

For clarity, the interpretation of FAMD output is demonstrated through a case study, examining the effects of independent chromatographic parameters on retention, resolution (reflected by PkC**), and peak distortion (characterized by peak skewness), and identifying compounds most vulnerable to changes in these parameters. Nevertheless, the presented statistical methods are universal and can be used for various other parameters and their combinations.

###  Chromatographic retention evaluation

**Fig. 1:**
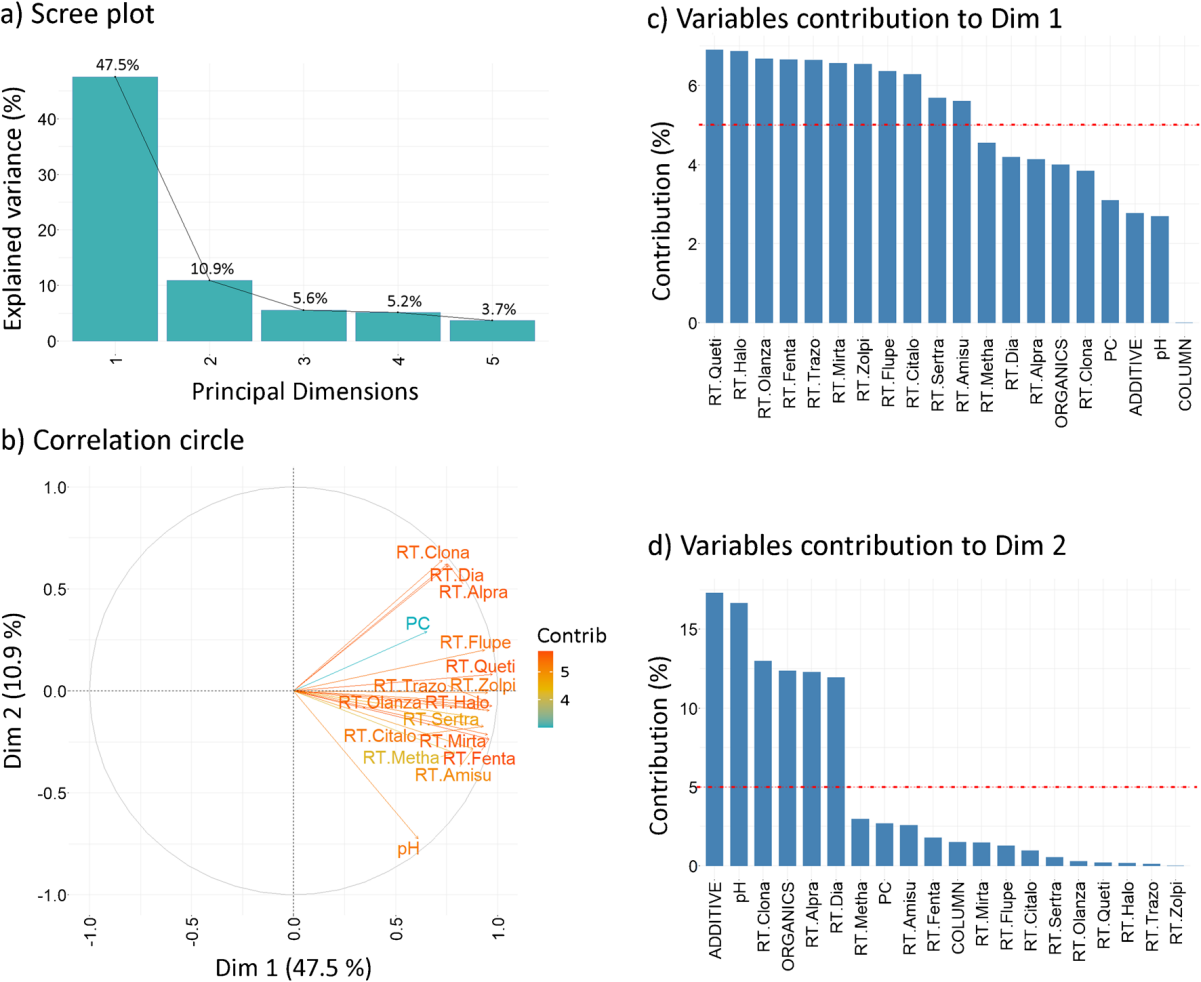
Retention evaluation. **a** Scree plot, **b** correlation circle, **c** variables’ contribution to principal dimension 1 (Dim 1) and **d** principal dimension 2 (Dim 2). The red dashed line in **c** and **d** indicates the expected mean contribution. Abbreviations: mobile phase additive (ADDITIVE), organic solvent (ORGANICS), stationary phase (COLUMN), retention time (RT), quetiapine (Queti), zolpidem (Zolpi), mirtazapine (Mirta), flupentixol (Flupe), trazodone (Trazo), sertraline (Sertra), alprazolam (Alpra), citalopram (Citalo), olanzapine (Olanza), amisulpride (Amisu), fentanyl (Fenta), clonazepam (Clona), methamphetamine (Metha), haloperidol (Halo), diazepam (Dia)

The scree plot (Fig. 1a) shows that the first two principal dimensions account for approximately 60% of the explained data variability, with Dim 1 contributing 47.5% and Dim 2 contributing 10.9%. Due to the considerably lower rate of variability change in higher principal dimensions, only Dim 1 and Dim 2 were selected for further interpretation.

The correlation circle (Fig. 1b) illustrates that vectors align primarily with the positive side of Dim 1, except for those representing clonazepam, alprazolam, diazepam, and pH, demonstrating their high correlation with both principal dimensions. A statistically significant strong positive correlation in retention times, confirmed with Spearman’s test (*p* < 0.05) (Suppl. Information, Table S3), was observed for clonazepam-diazepam-alprazolam (*r* > 0.937), trazodone-haloperidol-olanzapine (*r* > 0.930), and mirtazapine-fentanyl (*r* > 0.974), indicating closely related retention behaviors of these groups of compounds that respond similarly to variations in mobile phase composition and stationary phase.

Furthermore, retention times show a variable positive correlation with pH, affecting retention differently, with mirtazapine (*r* = 0.790) and flupentixol (*r* = 0.413) exhibiting the most and least pH sensitivity, respectively. The orthogonal orientation of the pH vector relative to the vectors of clonazepam, diazepam, and alprazolam (*r* < 0.063) points to the statistically insignificant effect (*p* > 0.05) of pH on the retention of these benzodiazepines. The insensitivity of these compounds to pH changes together with their high contribution to the explained variability implies that variables other than pH significantly impact their retention. These findings suggest that pH adjustment is an effective strategy for separation fine-tuning, except for clonazepam, diazepam, and alprazolam. The orthogonality of the PkC** vector with respect to pH (*r* = 0.204, *p* > 0.05) implies the minimal impact of pH on overall resolution. Thus, increasing pH generally increases retention; however, it minimally impacts overall resolution, as indicated by PkC** metrics.

The bar plots of variables’ contribution (Fig. 1c, d) illustrate that the variability along Dim 1 mainly originates from variations in retention times of compounds, while Dim 2 is significantly influenced by mobile phase additive, pH, and organic solvent, identifying them as key factors affecting retention characteristics. In contrast, the type of stationary phase shows minimal impact in both principal dimensions, indicating limited effects on retention. The plot further shows that except methamphetamine, all compounds are sensitive to changes in chromatography, according to their high contributions to Dim 1 and Dim 2.

**Fig. 2:**
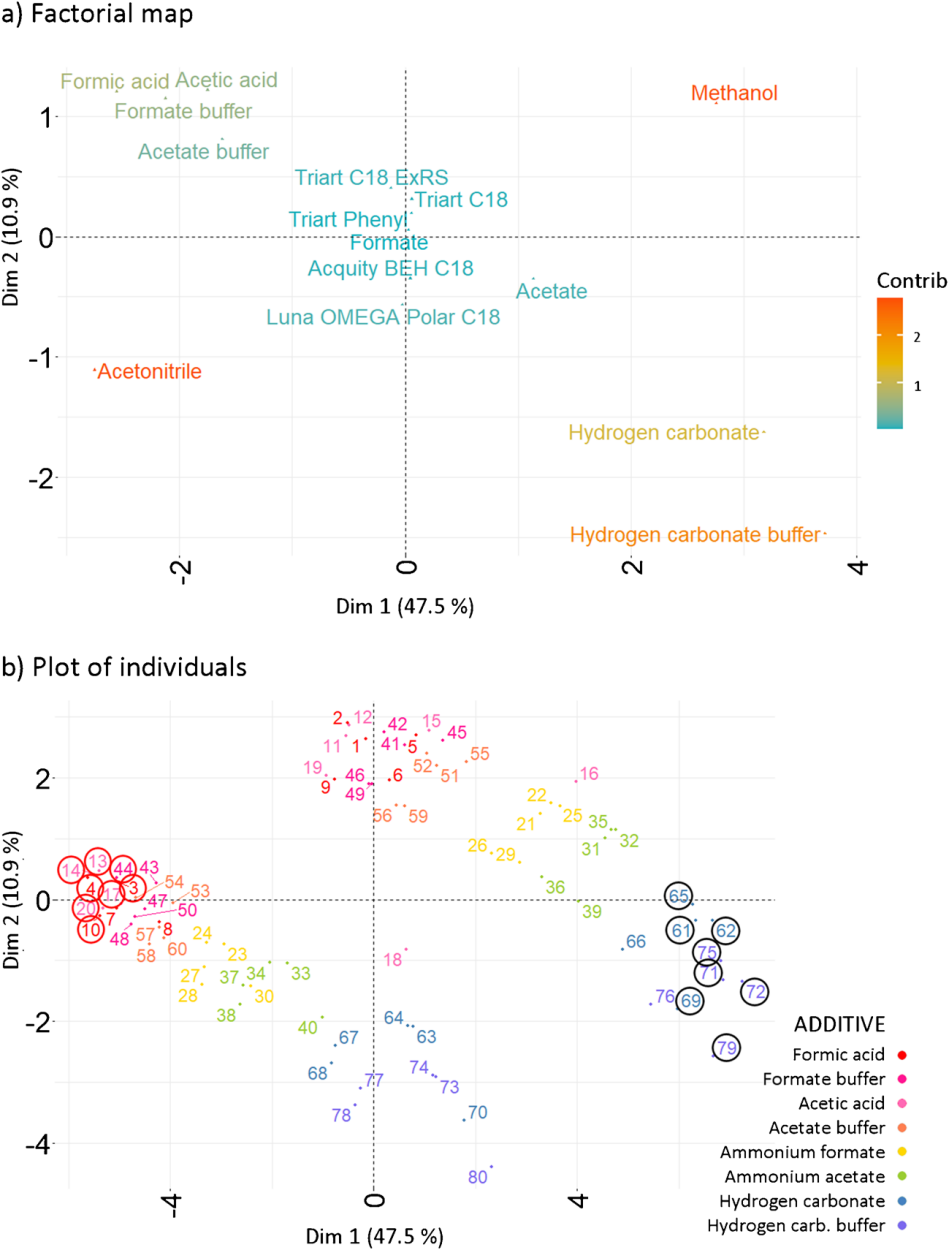
Retention evaluation. **a** Factorial map and **b** plot of individuals, projected onto the first two principal dimensions (Dim 1, Dim 2). The numerical annotations correspond to the individuals’ ID (Suppl. Information, Table S1). The circles highlight combinations giving the highest (black) and lowest (red) mean retention, based on the first and last deciles for the mean retention of compounds

The factorial map (Fig. 2a) shows significant influence of organic solvents and alkaline mobile phases on retention, based on their contribution to the explained variance. The positioning of methanol and acetonitrile at the opposing ends of Dim 1 and Dim 2 reflects their distinct impacts. Similarly, phases containing hydrogen carbonate, located in the lower-right quadrant, display opposite retention characteristics compared to other additives.

The positioning of Acquity BEH C18 and Luna OMEGA Polar C18 columns suggests that while they behave similarly, they influence retention differently from the Triart C18, Triart C18 ExRS, and Triart Phenyl.

Overall, the factorial map confirms that retention is more significantly influenced by variations in organic solvents and specific additives than by the stationary phases according to their minimal contribution to the dataset variance.

The plot of individuals (Fig. 2b) reveals the primary formation of two distinct clusters, corresponding to the distribution based on the type of organic solvent used (Suppl. Information, Fig. S1a). According to color-based coding, a secondary trend correlating with the type of mobile phase additive emerges. Combinations using alkaline additives cluster in the lower right, those with ammonium formate and acetate are centrally located, and combinations with acidic additives group in the upper left. The lack of a distinct pattern in the color-based coding according to the stationary phase highlights minimal influence of used stationary phase on retention characteristics (Suppl. Information, Fig. S1b). This supports the findings from other FAMD outputs. A retrospective analysis of the original dataset showed that the highest mean retention (1st decile) is related to individuals with IDs 72, 71, 75, 79, 62, 61, 69, and 65, while the lowest was observed for IDs 14, 4, 13, 20, 10, 44, 3, and 17 (10th decile). Mapping these individuals onto the plot of individuals reveals that combinations of chromatographic parameters leading to similar outcomes are positioned close together in the corresponding regions of the space defined by Dim 1 and Dim 2. Integrating insights from the factorial map, the plot of individuals, and the original dataset shows that mobile phases containing hydrogen carbonate and methanol correlate with higher mean retention times. Conversely, the use of acetonitrile combined with additives such as formic acid, acetic acid, ammonium formate buffer, or acetate buffer is linked to opposite outcomes. Additionally, the impact of the stationary phase on retention is less pronounced compared to other factors.

### Peak skewness evaluation

**Fig. 3:**
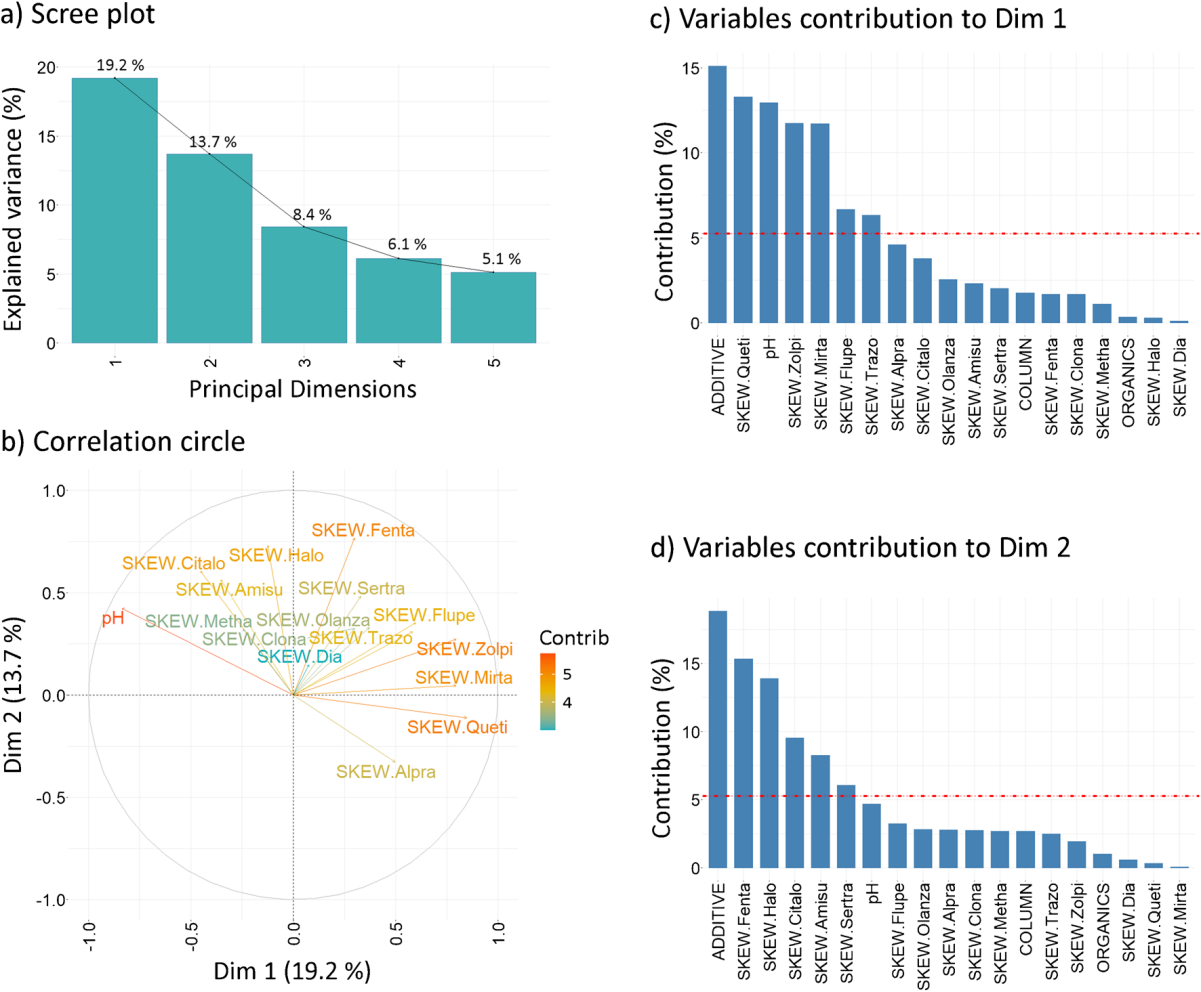
FAMD outputs in peak skewness (SKEW) evaluation. **a** Scree plot. **b** Correlation circle. **c** Variables’ contribution to principal dimension 1 (Dim 1) and **d** principal dimension 2 (Dim 2). The red dashed line in **c** and **d** indicates the expected mean contribution. For abbreviations, refer to Fig. 1

The scree plot (Fig. 3a) shows that the first two principal dimensions explain approximately 33% of the data variability, with Dim 1 accounting for 19.2% and Dim 2 for 13.7%. Following the “elbow criterion,” the third principal dimension, accounting for an additional 8.4% of variance, was included for evaluation in the plot of individuals. The lower variability explained in the first two principal dimensions may be attributed to the less pronounced effect of independent parameters on peak skewness, leading to higher noise in the data and distribution across the higher principal dimensions.

The correlation circle (Fig. 3b) indicates that the variability along Dim 1 is primarily influenced by the peak skewness of quetiapine, zolpidem, mirtazapine, and pH, while Dim 2 is mostly affected by fentanyl and haloperidol. In contrast, diazepam, clonazepam, olanzapine, and methamphetamine contribute the least to both principal dimensions, suggesting that their peak shapes are less impacted by changes in chromatographic parameters. Spearman’s correlation analysis (*p* < 0.05, Suppl. Information, Table S4) confirmed a strong positive correlation between pH and peak skewness of citalopram (*r* = 0.640) and a strong negative correlation with quetiapine (*r* =  − 0.750), indicating a significant dependence of their peak skewness on pH. Methamphetamine (*r* = 0.326), haloperidol (*r* = 0.427), and amisulpride (*r* = 0.575) exhibited weak to moderate positive correlations, while flupentixol (*r* =  − 0.224), alprazolam (*r* =  − 0.505), mirtazapine (*r* =  − 0.545), and zolpidem (*r* =  − 0.555) showed weak to moderate negative correlations. Very weak and statistically non-significant (*p* > 0.05) correlations were observed for trazodone, clonazepam, olanzapine, sertraline, diazepam, and fentanyl (*r* =  − 0.165 to 0.153). In summary, changes in pH significantly affected the peak skewness of citalopram, which increased with higher pH, and quetiapine, which showed the opposite effect. Other compounds, including trazodone, clonazepam, olanzapine, sertraline, diazepam, and fentanyl, were less responsive to pH fluctuations. Interestingly, the peak skewness of fentanyl, despite contributing significantly to the variability of the dataset, did not correlate with pH levels, suggesting that other factors influence its peak characteristics. The bar plots of variables’ contribution (Fig. 3c, d) show that the mobile phase additive and pH are the primary independent drivers of the variability along Dim 1, while the additive alone influences Dim 2. The peak skewness variation in Dim 1 is notably higher for quetiapine, zolpidem, mirtazapine, flupentixol, and trazodone, while fentanyl, haloperidol, citalopram, amisulpride, and sertraline primarily contribute to Dim 2. It indicates that the peak distortion of these compounds is sensitive to changes in stationary and mobile phase composition. The plot showed that the mobile phase additive is the key factor affecting peak skewness, with the type of stationary phase and the organic solvent playing minor roles. Unlike the correlation circle, this plot clarifies factors affecting fentanyl skewness, which primarily stems from the nature of the mobile phase additive, rather than from the pH or stationary phase used.

**Fig. 4:**
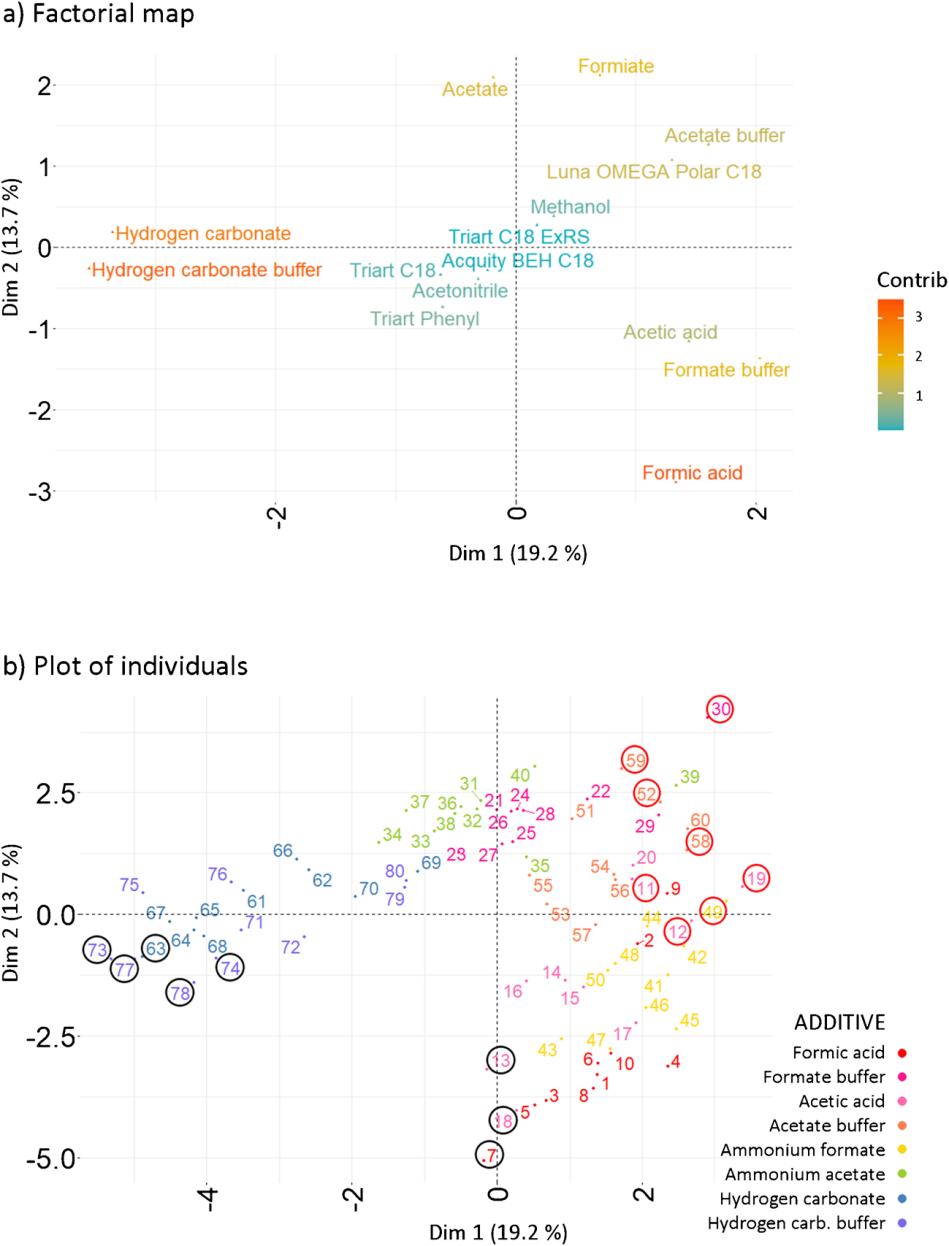
Evaluation of peak skewness. **a **Factorial map and **b** plot of individuals, projected onto the first two principal dimensions. The numerical annotations correspond to the individuals’ ID (Suppl. Information, Table S1). The circles highlight combinations giving optimal (black) and suboptimal (red) results, based on the first and last deciles for the absolute mean peak skewness

The factorial map (Fig. 4a) identifies hydrogen carbonate-containing phases and formic acid as key factors affecting peak skewness. On the map, the alkaline additives cluster to the left and the acidic ones to the right, indicating the correlation of Dim 1 with pH, while a pH-independent pattern is evident along Dim 2. The impact of additives such as acetic acid, ammonium acetate, acetate buffer, ammonium formate, or formate buffer on the peak skewness is less pronounced. The use of acetate buffer or the Luna OMEGA Polar C18 stationary phase results in similar effects on skewness. Grouping of acetic acid and formate buffer shows that they exert similar effects as well. The map also exhibits that the stationary phases such as Triart C18, Triart Phenyl, and Acquity BEH C18, along with acetonitrile, lead to similar outcomes, different from that of Triart C18 ExRS and methanol. However, according to the contribution value, the overall influence of stationary phases and solvents is minimal, with the exception of the Luna OMEGA Polar C18, diverging from the other stationary phases.

The plot of individuals (Fig. 4b) shows a distribution forming cluster on the right, with two tails pointing to the lower left. The plot also shows a pattern according to the mobile phase additive. Incorporating the third principal dimension into the evaluation reveals that alkaline additives form a separate cluster in the 3D space (Suppl. Information, Fig. S3). Color coding by organic solvent or stationary phase (Suppl. Information, Fig. S2a, S2b) reveals no discernible pattern, indicating a random distribution of individuals according to these factors and corroborating their negligible effect on peak skewness. A retrospective analysis of the original dataset showed the lowest mean absolute peak skewness in individuals corresponding to IDs 7, 18, 78, 77, 73, 63, 74, and 13 (1st decile), while the highest correlates with IDs 30, 19, 11, 49, 59, 52, 58, and 12 (10th decile). A summary of the available data indicates that optimal results stem from using hydrogen carbonate or formic acid-containing phases, Triart C18, Triart Phenyl, or Acquity BEH C18 as stationary phases, and acetonitrile as the solvent. Conversely, high peak distortion is very strongly associated with the use of Luna OMEGA Polar C18, acetate buffer, or ammonium formate.

### Hierarchical clustering evaluation

**Fig. 5:**
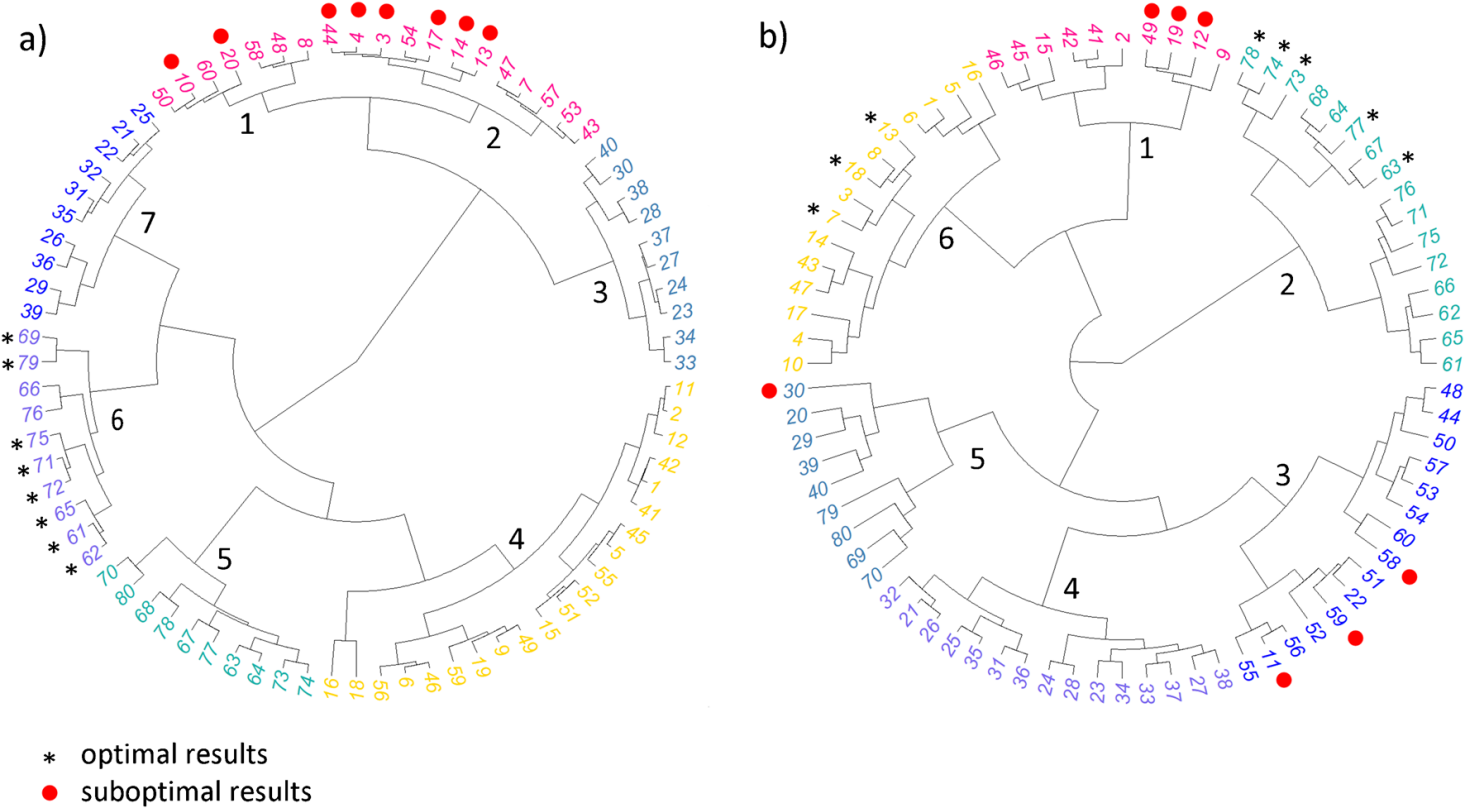
Dendrograms based on FAMD results to assess similarity among individuals in the investigation of factors influencing **a** retention behavior and **b** peak skewness. Individuals giving optimal (black asterisk *) and suboptimal (red dot  ) results

The HC dendrogram (Fig. 5) showed that the distance on the plot of individuals (Fig. 4b, Suppl. Information, Fig. S3) may not fully capture the variability across other principal dimensions. Using the full range of principal dimensions from FAMD, the HC provided a more comprehensive representation for similarity assessment and helped to identify combinations of chromatographic parameters that yield the most related results. In investigating retention behavior via HC, the output dendrogram was segmented into six groups (Fig. 5a), each characterized by a unique combination of chromatographic parameters (Suppl. Information, Table S5). The clustering in the dendrogram was correlated with the results obtained by plot of individuals (Fig. 2b).

For example, the combinations yielding the highest mean retention were present in cluster No. 6, which corresponded to the use of the alkaline mobile phases and methanol as the solvent. Conversely, the lowest mean retention was observed in clusters No. 1 and 2, typically linked to acidic additives, acetonitrile, or the Luna OMEGA Polar C18 column.

The peak skewness investigation revealed that IDs representing optimal or suboptimal conditions are distributed across multiple clusters (Fig. 5b), indicating that a set of more diverse independent parameters can lead to similar outcomes. This finding aligns well with the distribution observed in Fig. 4b, where, in particular, individuals producing lower mean values of peak skewness exhibited a wider distribution across the principal dimensions Dim 1 and Dim 2.

### Summary of data analysis

The results suggest that combinations of chromatographic parameters leading to low peak distortion tend to result in reduced retention times and vice versa, indicating that the selection of chromatography parameters necessitates a balance between these opposing outcomes. Since there was no overlap between the two groups, further evaluation was undertaken. It was determined that retention should be prioritized over peak distortion at this stage, as variations in peak skewness were less pronounced in the dataset compared to differences in retention. The data analysis revealed that the use of methanol and hydrogen carbonate buffer was the factor contributing to higher retention. Based on this, the skewness data table was sorted by increasing overall peak skewness, and the first entry utilizing methanol as the solvent and hydrogen carbonate buffer as the additive was selected. This corresponded to record ID 71, which exhibited the second-highest mean retention and was classified within the cluster associated with low overall peak distortion (Figs. 4b and 5b). This combination represents an effective compromise between achieving higher retention and maintaining peak shape integrity.

The *K*-fold cross-validation showed good stability of the FAMD method as demonstrated by only insignificant changes in the coordinates of individuals (Suppl. Information, Fig. S4) and variables (Suppl. Information, Fig. S5) in Dim 1 and Dim 2. This indicates that the obtained groupings and trends are neither random nor related to the particular structure of the dataset.

### Flow rate and temperature optimization

**Fig. 6:**
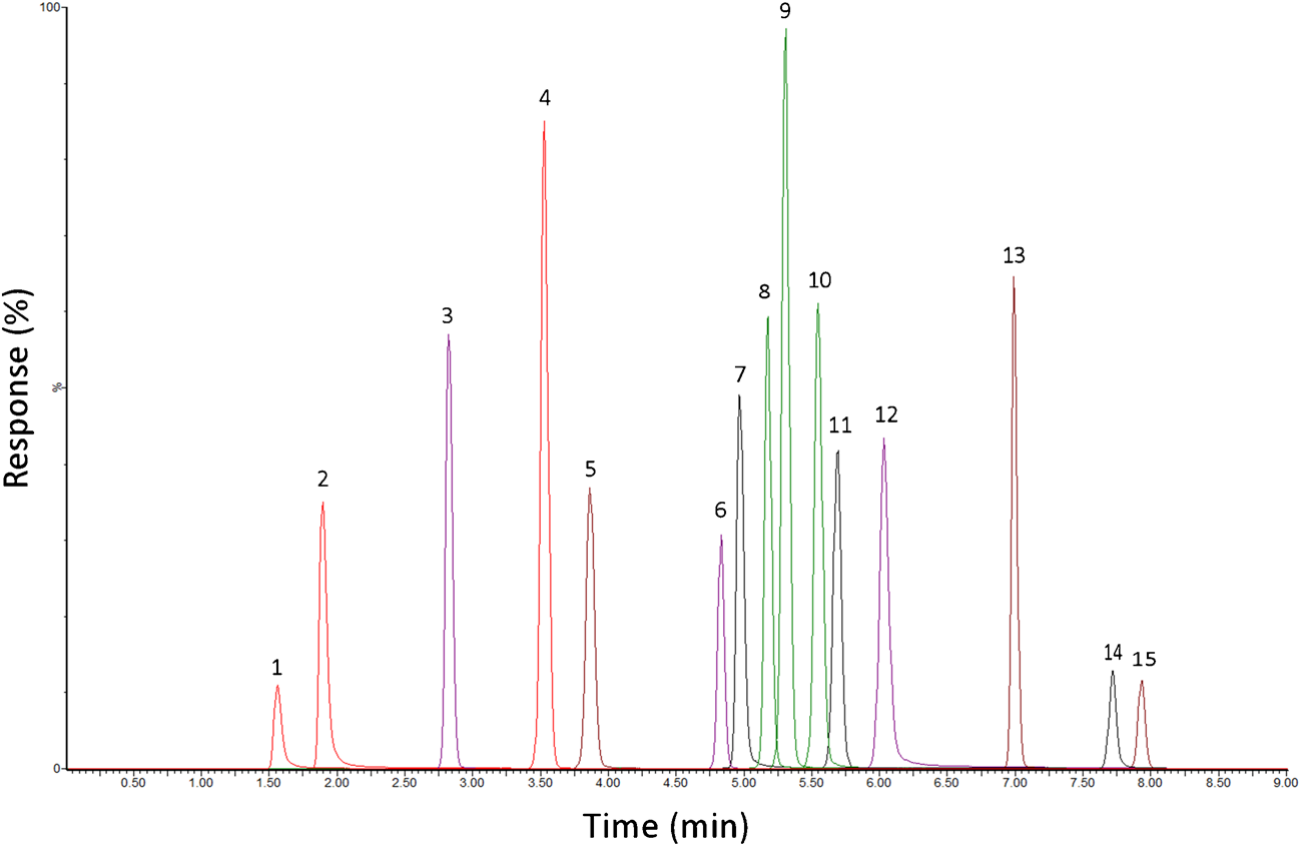
Chromatogram corresponding to optimal combination of independent chromatographic parameters (“Samples and solutions” — optimized gradient separation) obtained through FAMD, HC, and BBD analysis. The compounds ID 1: methamphetamine, 2: amisulpride, 3: clonazepam, 4: alprazolam, 5: zolpidem, 6: diazepam, 7: olanzapine, 8: citalopram, 9: mirtazapine, 10: quetiapine, 11: trazadone, 12: haloperidol, 13: fentanyl, 14: flupentixol, and 15: sertraline

The initial screening experiment, subjected to FAMD analysis, was conducted using a generic monolinear gradient at a temperature of 40 °C and a flow rate of 0.35 mL/min. Although the combination of chromatographic parameters identified under ID 71 offered sufficient retention of compounds and satisfactory peak shapes, the separation of critical pairs olanzapine-mirtazapine, quetiapine-trazodone, and sertraline-flupentixol was suboptimal. Despite these compounds having different MRM transitions, allowing for their analysis using a mass spectrometer detector, efforts were focused on achieving the best possible separation in the case that a non-selective detector, such as UV–VIS, would be used for analysis. Consequently, the generic gradient was modified to an optimized gradient, which resolved the separation of most compounds except the olanzapine-mirtazapine. To resolve these compounds, the optimization of temperature and flow rate was employed, using BBD optimization method. Contour plot and response surface plot resulting from the optimization are presented in Fig. S6 (Suppl. Information). Based on the results, the optimal separation of the critical pair was achieved at temperature of 60 °C and a flow rate of 0.3 mL/min. The resulting chromatogram is presented in Fig. 6.

## Conclusions

A novel methodology was employed for the optimization data evaluation in development of liquid chromatography-mass spectrometry separation method for 15 compounds affecting the central nervous system. Through the use of exploratory multivariate analysis, specifically factor analysis of mixed data and hierarchical clustering via R Language script, the data evaluation was significantly streamlined. This approach facilitated a better understanding of the effect of selected qualitative and quantitative parameters influencing separation of investigated compounds while reducing subjectivity in data assessment. Furthermore, factor analysis of mixed data and hierarchical clustering provided a straightforward way to compare separations based on different chromatographic conditions, facilitating the evaluation of similarity and transition to a different setting when change in chromatography is required. The insights gained from the methodology can be used in guiding future development stages. Additionally, the R Language script developed for statistical analysis, along with the ChromaFAMDeX application are freely available for use.

## Supplementary Information

Below is the link to the electronic supplementary material.

Supplementary file1 (DOCX 1049 KB)
