# Supplementary material for: Exploratory multivariate analysis using R Language for method development in liquid chromatography
Source: Anal Bioanal Chem. 2025 Jan 10;417(6):1113–25. doi: 10.1007/s00216-024-05705-y (PMC11802592; doi:10.1007/s00216-024-05705-y)
Supplement: Supplementary file 1 — Supplementary file1 (DOCX 1049 KB) [file 216_2024_5705_MOESM1_ESM.docx]

**Supplementary data**

**Exploratory Multivariate Analysis using R Language for Method Development in Liquid Chromatography**

Miloš Hroch

ORCID ID: 0000-0002-5583-2942

*Charles University, Faculty of Medicine in Hradec Králové, Department of Medical Biochemistry, Šimkova 870, 500 03 Hradec Králové, Czech Republic*

**Corresponding author:** Miloš Hroch, Tel.: +420495816340,

E-mail address: [hrochm@lfhk.cuni.cz](mailto:hrochm@lfhk.cuni.cz)

**Table of Contents**

[1. Tables and Figures 3](#_Toc183096385)

[Table S1 3](#_Toc183096386)

[Table S2 5](#_Toc183096387)

[Table S3 6](#_Toc183096388)

[Table S4 7](#_Toc183096389)

[Table S5 8](#_Toc183096390)

[Table S6 9](#_Toc183096391)

[Table S7 10](#_Toc183096392)

[Fig. S1 11](#_Toc183096393)

[Fig. S2 12](#_Toc183096394)

[Fig. S3 13](#_Toc183096395)

[Fig. S4 14](#_Toc183096396)

[Fig. S5 15](#_Toc183096397)

[Fig. S6 16](#_Toc183096398)

[2. Preparation of Data for Processing by R Language 17](#_Toc183096399)

[Dataset Structure 17](#_Toc183096400)

[Dataset Preparation and Export 17](#_Toc183096401)

[Dataset for ChromaFAMDeX 17](#_Toc183096402)

[3. R Language Script to Perform FAMD and HC Analysis 18](#_Toc183096403)

[4. R Language Script to Perform Normality Test and Spearman’s Correlation Analysis 26](#_Toc183096404)

[5. R Language Script to Perform K-fold cross-validation 29](#_Toc183096405)

[6. Box-Behnken design optimization 32](#_Toc183096406)

# Tables and Figures

Table S1 List of stationary and mobile phases with the corresponding ID numbers

| **ID** | **COLUMN** | **Solvent A** | **Solvent B** | **Additive to solvent A and B*** | **pH**** |  | **ID** | **COLUMN** | **Solvent A** | **Solvent B** | **Additive to solvent A and B*** | **pH**** |
| --- | --- | --- | --- | --- | --- | --- | --- | --- | --- | --- | --- | --- |
| 1 | Triart C18 | water | methanol | 0.1% (v/v) formic acid | 2.74 |  | 21 | Triart C18 | water | methanol | 5 mM ammonium formate | 5.99 |
| 2 | Triart C18 ExRS |  |  |  |  |  | 22 | Triart C18 ExRS |  |  |  |  |
| 3 | Triart C18 | water | acetonitrile |  |  |  | 23 | Triart C18 | water | acetonitrile |  |  |
| 4 | Triart C18 ExRS |  |  |  |  |  | 24 | Triart C18 ExRS |  |  |  |  |
| 5 | Triart Phenyl | water | methanol |  |  |  | 25 | Triart Phenyl | water | methanol |  |  |
| 6 | Acquity BEH C18 |  |  |  |  |  | 26 | Acquity BEH C18 |  |  |  |  |
| 7 | Triart Phenyl | water | acetonitrile |  |  |  | 27 | Triart Phenyl | water | acetonitrile |  |  |
| 8 | Acquity BEH C18 |  |  |  |  |  | 28 | Acquity BEH C18 |  |  |  |  |
| 9 | Luna OMEGA Polar C18 | water | methanol |  |  |  | 29 | Luna OMEGA Polar C18 | water | methanol |  |  |
| 10 | Luna OMEGA Polar C18 | water | acetonitrile |  |  |  | 30 | Luna OMEGA Polar C18 | water | acetonitrile |  |  |
| 11 | Triart C18 | water | methanol | 0.1% (v/v) acetic acid | 3.34 |  | 31 | Triart C18 | water | methanol | 5 mM ammonium acetate | 6.61 |
| 12 | Triart C18 ExRS |  |  |  |  |  | 32 | Triart C18 ExRS |  |  |  |  |
| 13 | Triart C18 | water | acetonitrile |  |  |  | 33 | Triart C18 | water | acetonitrile |  |  |
| 14 | Triart C18 ExRS |  |  |  |  |  | 34 | Triart C18 ExRS |  |  |  |  |
| 15 | Triart Phenyl | water | methanol |  |  |  | 35 | Triart Phenyl | water | methanol |  |  |
| 16 | Acquity BEH C18 |  |  |  |  |  | 36 | Acquity BEH C18 |  |  |  |  |
| 17 | Triart Phenyl | water | acetonitrile |  |  |  | 37 | Triart Phenyl | water | acetonitrile |  |  |
| 18 | Acquity BEH C18 |  |  |  |  |  | 38 | Acquity BEH C18 |  |  |  |  |
| 19 | Luna OMEGA Polar C18 | water | methanol |  |  |  | 39 | Luna OMEGA Polar C18 | water | methanol |  |  |
| 20 | Luna OMEGA Polar C18 | water | acetonitrile |  |  |  | 40 | Luna OMEGA Polar C18 | water | acetonitrile |  |  |

**Table S1** Continuation …

| **ID** | **COLUMN** | **Solvent A** | **Solvent B*** | **Additive to solvent A and B** | **pH**** |  | **ID** | **COLUMN** | **Solvent A** | **Solvent B*** | **Additive to solvent A and B** | **pH**** |
| --- | --- | --- | --- | --- | --- | --- | --- | --- | --- | --- | --- | --- |
| 41 | Triart C18 | water | methanol | 5 mM ammonium formate, 0.1% (v/v) formic acid | 3.09 |  | 61 | Triart C18 | water | methanol | 5 mM ammonium hydrogen carbonate | 7.94 |
| 42 | Triart C18 ExRS |  |  |  |  |  | 62 | Triart C18 ExRS |  |  |  |  |
| 43 | Triart C18 | water | acetonitrile |  |  |  | 63 | Triart C18 | water | acetonitrile |  |  |
| 44 | Triart C18 ExRS |  |  |  |  |  | 64 | Triart C18 ExRS |  |  |  |  |
| 45 | Triart Phenyl | water | methanol |  |  |  | 65 | Triart Phenyl | water | methanol |  |  |
| 46 | Acquity BEH C18 |  |  |  |  |  | 66 | Acquity BEH C18 |  |  |  |  |
| 47 | Triart Phenyl | water | acetonitrile |  |  |  | 67 | Triart Phenyl | water | acetonitrile |  |  |
| 48 | Acquity BEH C18 |  |  |  |  |  | 68 | Acquity BEH C18 |  |  |  |  |
| 49 | Luna OMEGA Polar C18 | water | methanol |  |  |  | 69 | Luna OMEGA Polar C18 | water | methanol |  |  |
| 50 | Luna OMEGA Polar C18 | water | acetonitrile |  |  |  | 70 | Luna OMEGA Polar C18 | water | acetonitrile |  |  |
| 51 | Triart C18 | water | methanol | 5 mM ammonium acetate, 0.1% (v/v) acetic acid | 4.21 |  | 71 | Triart C18 | water | methanol | 5 mM ammonium hydrogen carbonate, 0.02% ammonia | 9.21 |
| 52 | Triart C18 ExRS |  |  |  |  |  | 72 | Triart C18 ExRS |  |  |  |  |
| 53 | Triart C18 | water | acetonitrile |  |  |  | 73 | Triart C18 | water | acetonitrile |  |  |
| 54 | Triart C18 ExRS |  |  |  |  |  | 74 | Triart C18 ExRS |  |  |  |  |
| 55 | Triart Phenyl | water | methanol |  |  |  | 75 | Triart Phenyl | water | methanol |  |  |
| 56 | Acquity BEH C18 |  |  |  |  |  | 76 | Acquity BEH C18 |  |  |  |  |
| 57 | Triart Phenyl | water | acetonitrile |  |  |  | 77 | Triart Phenyl | water | acetonitrile |  |  |
| 58 | Acquity BEH C18 |  |  |  |  |  | 78 | Acquity BEH C18 |  |  |  |  |
| 59 | Luna OMEGA Polar C18 | water | methanol |  |  |  | 79 | Luna OMEGA Polar C18 | water | methanol |  |  |
| 60 | Luna OMEGA Polar C18 | water | acetonitrile |  |  |  | 80 | Luna OMEGA Polar C18 | water | acetonitrile |  |  |

** Solvent B was composed of the corresponding organic solvent at a concentration of 95 % (v/v) in water*

*** pH of the Solvent A*

Table S2 List of analyzed compounds, quantifier and qualifier MRM transitions, collision energies, and ion source settings

| **Therapeutic group** | **Compound** | **Quantifier MRM** | **Qualifier MRM** | **Collision energy (eV)** |
| --- | --- | --- | --- | --- |
| Antidepressants | Mirtazapine | 266.1 - 195.1 | 266.1 - 72.2 | 25 |
|  | Sertraline | 306.1 - 275.1 | 306.1 - 159.0 | 15 |
|  | Citalopram | 325.1 - 109.0 | 325.1 - 262.0 | 25 |
|  | Trazodone | 372.1 - 176.1 | 372.1 - 148.1 | 25 |
|  |  |  |  |  |
| Antipsychotics | Olanzapine | 313.2 - 256.2 | 313.2 - 84.0 | 22 |
|  | Amisulpride | 369.9 - 242.2 | 369.9 - 112.0 | 30 |
|  | Haloperidol | 376.1 - 165.1 | 376.1 - 123.1 | 22 |
|  | Quetiapine | 384.1 - 253.0 | 384.1 - 279.1 | 22 |
|  | Flupentixol | 435.3 - 128.1 | 435.3 - 305.0 | 25 |
|  |  |  |  |  |
| Benzodiazepines | Diazepam | 285.1 - 193.2 | 285.1 - 154.0 | 30 |
|  | Zolpidem | 308.1 - 263.0 | 308.1 - 235.1 | 25 |
|  | Alprazolam | 309.1 - 281.0 | 309.1 - 165.0 | 25 |
|  | Clonazepam | 316.0 - 270.1 | 316.0 - 214.0 | 25 |
|  |  |  |  |  |
| Drugs of abuse | Methamphetamine | 150.1 - 119.0 | 150.1 - 91.1 | 15 |
|  | Fentanyl | 337.2 - 188.1 | 337.2 - 105.1 | 22 |
|  |  |  |  |  |
| **Mass spectrometer settings** |  |  |  |  |
| Capillary Voltage | 0.5 kV |  |  |  |
| Cone Voltage | 40 V |  |  |  |
| Desolvation Gas Flow | 1000 L/h |  |  |  |
| Desolvation Gas Temperature | 600 °C |  |  |  |

Table S3 Spearman’s correlation coefficients calculated to assess the relations among numerical variables in investigation of retention behavior

| **r** | **pH** | **RT.Citalo** | **RT.Trazo** | **RT.Mirta** | **RT.Sertra** | **RT.Metha** | **RT.Fenta** | **RT.Olanza** | **RT.Amisu** | **RT.Queti** | **RT.Halo** | **RT.Flupe** | **RT.Zolpi** | **RT.Clona** | **RT.Alpra** | **RT.Dia** | **PC**** |
| --- | --- | --- | --- | --- | --- | --- | --- | --- | --- | --- | --- | --- | --- | --- | --- | --- | --- |
| **pH** | - | 0.584 | 0.676 | 0.790 | 0.603 | 0.570 | 0.727 | 0.692 | 0.615 | 0.515 | 0.596 | 0.416 | 0.646 | 0.063 | 0.115 | 0.171 | 0.205 |
| **RT.Citalo** | 0.584 | - | 0.892 | 0.886 | 0.962 | 0.969 | 0.935 | 0.921 | 0.958 | 0.907 | 0.984 | 0.888 | 0.873 | 0.567 | 0.631 | 0.614 | 0.658 |
| **RT.Trazo** | 0.676 | 0.892 | - | 0.970 | 0.882 | 0.877 | 0.964 | 0.981 | 0.867 | 0.952 | 0.930 | 0.898 | 0.982 | 0.628 | 0.666 | 0.692 | 0.672 |
| **RT.Mirta** | 0.790 | 0.886 | 0.970 | - | 0.887 | 0.884 | 0.974 | 0.975 | 0.877 | 0.909 | 0.918 | 0.855 | 0.946 | 0.514 | 0.571 | 0.601 | 0.608 |
| **RT.Sertra** | 0.603 | 0.962 | 0.882 | 0.887 | - | 0.944 | 0.945 | 0.891 | 0.891 | 0.906 | 0.978 | 0.893 | 0.841 | 0.471 | 0.533 | 0.561 | 0.721 |
| **RT.Metha** | 0.570 | 0.969 | 0.877 | 0.884 | 0.944 | - | 0.911 | 0.905 | 0.924 | 0.910 | 0.963 | 0.913 | 0.867 | 0.564 | 0.635 | 0.627 | 0.651 |
| **RT.Fenta** | 0.727 | 0.935 | 0.964 | 0.974 | 0.945 | 0.911 | - | 0.971 | 0.917 | 0.923 | 0.964 | 0.877 | 0.929 | 0.535 | 0.583 | 0.610 | 0.664 |
| **RT.Olanza** | 0.692 | 0.921 | 0.981 | 0.975 | 0.891 | 0.905 | 0.971 | - | 0.920 | 0.937 | 0.940 | 0.892 | 0.967 | 0.631 | 0.665 | 0.667 | 0.630 |
| **RT.Amisu** | 0.615 | 0.958 | 0.867 | 0.877 | 0.891 | 0.924 | 0.917 | 0.920 | - | 0.850 | 0.932 | 0.822 | 0.861 | 0.600 | 0.645 | 0.607 | 0.550 |
| **RT.Queti** | 0.515 | 0.907 | 0.952 | 0.909 | 0.906 | 0.910 | 0.923 | 0.937 | 0.850 | - | 0.947 | 0.978 | 0.948 | 0.713 | 0.751 | 0.776 | 0.752 |
| **RT.Halo** | 0.596 | 0.984 | 0.930 | 0.918 | 0.978 | 0.963 | 0.964 | 0.940 | 0.932 | 0.947 | - | 0.928 | 0.906 | 0.592 | 0.648 | 0.657 | 0.714 |
| **RT.Flupe** | 0.416 | 0.888 | 0.898 | 0.855 | 0.893 | 0.913 | 0.877 | 0.892 | 0.822 | 0.978 | 0.928 | - | 0.894 | 0.719 | 0.766 | 0.787 | 0.762 |
| **RT.Zolpi** | 0.646 | 0.873 | 0.982 | 0.946 | 0.841 | 0.867 | 0.929 | 0.967 | 0.861 | 0.948 | 0.906 | 0.894 | - | 0.670 | 0.707 | 0.719 | 0.640 |
| **RT.Clona** | 0.063 | 0.567 | 0.628 | 0.514 | 0.471 | 0.564 | 0.535 | 0.631 | 0.600 | 0.713 | 0.592 | 0.719 | 0.670 | - | 0.947 | 0.937 | 0.550 |
| **RT.Alpra** | 0.115 | 0.631 | 0.666 | 0.571 | 0.533 | 0.635 | 0.583 | 0.665 | 0.645 | 0.751 | 0.648 | 0.766 | 0.707 | 0.947 | - | 0.956 | 0.587 |
| **RT.Dia** | 0.171 | 0.614 | 0.692 | 0.601 | 0.561 | 0.627 | 0.610 | 0.667 | 0.607 | 0.776 | 0.657 | 0.787 | 0.719 | 0.937 | 0.956 | - | 0.659 |
| **PC**** | 0.205 | 0.658 | 0.672 | 0.608 | 0.721 | 0.651 | 0.664 | 0.630 | 0.550 | 0.752 | 0.714 | 0.762 | 0.640 | 0.550 | 0.587 | 0.659 | - |

Table S4 Spearman’s correlation coefficients calculated to assess the relations among numerical variables in the investigation of peak skewness

| **r** | pH | Skew.Citalo | Skew.Trazo | Skew.Mirta | Skew.Sertra | Skew.Metha | Skew.Fenta | Skew.Olanza | Skew.Amisu | Skew.Queti | Skew.Halo | Skew.Flupe | Skew.Zolpi | Skew.Clona | Skew.Alpra | Skew.Dia |
| --- | --- | --- | --- | --- | --- | --- | --- | --- | --- | --- | --- | --- | --- | --- | --- | --- |
| pH | - | 0.640 | -0.165 | -0.545 | -0.085 | 0.326 | 0.153 | -0.119 | 0.575 | -0.750 | 0.427 | -0.224 | -0.555 | -0.155 | -0.505 | 0.048 |
| Skew.Citalo | 0.640 | - | -0.035 | -0.391 | 0.155 | 0.163 | 0.408 | 0.012 | 0.521 | -0.356 | 0.463 | -0.050 | -0.198 | 0.152 | -0.297 | 0.291 |
| Skew.Trazo | -0.165 | -0.035 | - | 0.404 | 0.352 | -0.004 | 0.349 | 0.255 | -0.116 | 0.391 | 0.054 | 0.287 | 0.501 | 0.248 | 0.094 | 0.144 |
| Skew.Mirta | -0.545 | -0.391 | 0.404 | - | 0.081 | -0.140 | 0.213 | 0.374 | -0.227 | 0.661 | -0.182 | 0.421 | 0.677 | 0.309 | 0.227 | 0.054 |
| Skew.Sertra | -0.085 | 0.155 | 0.352 | 0.081 | - | 0.132 | 0.476 | 0.147 | 0.157 | 0.222 | 0.220 | 0.247 | 0.350 | 0.187 | 0.001 | 0.093 |
| Skew.Metha | 0.326 | 0.163 | -0.004 | -0.140 | 0.132 | - | 0.152 | -0.068 | 0.331 | -0.244 | 0.213 | 0.003 | 0.035 | -0.420 | -0.653 | -0.391 |
| Skew.Fenta | 0.153 | 0.408 | 0.349 | 0.213 | 0.476 | 0.152 | - | 0.411 | 0.238 | 0.211 | 0.420 | 0.362 | 0.439 | 0.274 | -0.006 | 0.311 |
| Skew.Olanza | -0.119 | 0.012 | 0.255 | 0.374 | 0.147 | -0.068 | 0.411 | - | -0.097 | 0.319 | 0.151 | 0.480 | 0.363 | 0.276 | 0.113 | 0.090 |
| Skew.Amisu | 0.575 | 0.521 | -0.116 | -0.227 | 0.157 | 0.331 | 0.238 | -0.097 | - | -0.331 | 0.462 | 0.008 | -0.191 | -0.043 | -0.355 | 0.058 |
| Skew.Queti | -0.750 | -0.356 | 0.391 | 0.661 | 0.222 | -0.244 | 0.211 | 0.319 | -0.331 | - | -0.116 | 0.352 | 0.735 | 0.375 | 0.413 | 0.143 |
| Skew.Halo | 0.427 | 0.463 | 0.054 | -0.182 | 0.220 | 0.213 | 0.420 | 0.151 | 0.462 | -0.116 | - | 0.207 | -0.012 | 0.128 | -0.110 | 0.199 |
| Skew.Flupe | -0.224 | -0.050 | 0.287 | 0.421 | 0.247 | 0.003 | 0.362 | 0.480 | 0.008 | 0.352 | 0.207 | - | 0.435 | 0.270 | 0.018 | 0.160 |
| Skew.Zolpi | -0.555 | -0.198 | 0.501 | 0.677 | 0.350 | 0.035 | 0.439 | 0.363 | -0.191 | 0.735 | -0.012 | 0.435 | - | 0.367 | 0.234 | 0.113 |
| Skew.Clona | -0.155 | 0.152 | 0.248 | 0.309 | 0.187 | -0.420 | 0.274 | 0.276 | -0.043 | 0.375 | 0.128 | 0.270 | 0.367 | - | 0.442 | 0.649 |
| Skew.Alpra | -0.505 | -0.297 | 0.094 | 0.227 | 0.001 | -0.653 | -0.006 | 0.113 | -0.355 | 0.413 | -0.110 | 0.018 | 0.234 | 0.442 | - | 0.453 |
| Skew.Dia | 0.048 | 0.291 | 0.144 | 0.054 | 0.093 | -0.391 | 0.311 | 0.090 | 0.058 | 0.143 | 0.199 | 0.160 | 0.113 | 0.649 | 0.453 | - |

**Abbreviations:** Retention time (RT), peak skewness (SKEW), quetiapine (Queti), zolpidem (Zolpi), mirtazapine (Mirta), flupentixol (Flupe), trazodone (Trazo), sertraline (Sertra), alprazolam (Alpra), citalopram (Citalo), olanzapine (Olanza), amisulpride (Amisu), fentanyl (Fenta), clonazepam (Clona), methamphetamine (Metha), haloperidol (Halo), and diazepam (Dia)

Table S5 Frequency of association of independent chromatographic parameters (SOLVENT, ADDITIVE, COLUMN) with clusters according to HC analysis on FAMD individuals. Frequencies are expressed as percentages (%) of each variable's occurrence within the clusters. Values exhibiting a frequency greater than 20% are highlighted

| **CLUSTER** | **1** | **2** | **3** | **4** | **5** | **6** | **7** |
| --- | --- | --- | --- | --- | --- | --- | --- |
|  | *Retention evaluation* | | | | | | |
| Methanol | 0.0 | 0.0 | 0.0 | 95.2 | 0.0 | 100.0 | 100.0 |
| Acetonitrile | 100.0 | 100.0 | 100.0 | 4.8 | 100.0 | 0.0 | 0.0 |
|  |  |  |  |  |  |  |  |
| Formic acid | 28.6 | 25.0 | 0.0 | 23.8 | 0.0 | 0.0 | 0.0 |
| Acetic acid | 14.3 | 25.0 | 0.0 | 28.6 | 0.0 | 0.0 | 0.0 |
| Formate buffer | 28.6 | 25.0 | 0.0 | 23.8 | 0.0 | 0.0 | 0.0 |
| Acetate buffer | 28.6 | 25.0 | 0.0 | 23.8 | 0.0 | 0.0 | 0.0 |
| Formate | 0.0 | 0.0 | 50.0 | 0.0 | 0.0 | 0.0 | 50.0 |
| Acetate | 0.0 | 0.0 | 50.0 | 0.0 | 0.0 | 0.0 | 50.0 |
| Hydrogen carbonate | 0.0 | 0.0 | 0.0 | 0.0 | 50.0 | 50.0 | 0.0 |
| Hydrogen carbonate buffer | 0.0 | 0.0 | 0.0 | 0.0 | 50.0 | 50.0 | 0.0 |
|  |  |  |  |  |  |  |  |
| Triart C18 | 0.0 | 33.3 | 20.0 | 19.0 | 20.0 | 20.0 | 20.0 |
| Triart C18 ExRS | 0.0 | 33.3 | 20.0 | 19.0 | 20.0 | 20.0 | 20.0 |
| Triart Phenyl | 0.0 | 33.3 | 20.0 | 19.0 | 20.0 | 20.0 | 20.0 |
| Acquity BEH C18 | 42.9 | 0.0 | 20.0 | 23.8 | 20.0 | 20.0 | 20.0 |
| Luna OMEGA Polar C18 | 57.1 | 0.0 | 20.0 | 19.0 | 20.0 | 20.0 | 20.0 |
|  | *Peak skewness evaluation* | | | | | | |
| Methanol | 100.0 | 50.0 | 46.7 | 46.7 | 44.4 | 26.7 | - |
| Acetonitrile | 0.0 | 50.0 | 53.3 | 53.3 | 55.6 | 73.3 | - |
|  |  |  |  |  |  |  |  |
| Formic acid | 20.0 | 0.0 | 0.0 | 0.0 | 0.0 | 53.3 | - |
| Acetic acid | 30.0 | 0.0 | 6.7 | 0.0 | 11.1 | 33.3 | - |
| Formate buffer | 50.0 | 0.0 | 20.0 | 0.0 | 0.0 | 13.3 | - |
| Acetate buffer | 0.0 | 0.0 | 66.7 | 0.0 | 0.0 | 0.0 | - |
| Formate | 0.0 | 0.0 | 6.7 | 46.7 | 22.2 | 0.0 | - |
| Acetate | 0.0 | 0.0 | 0.0 | 53.3 | 22.2 | 0.0 | - |
| Hydrogen carbonate | 0.0 | 50.0 | 0.0 | 0.0 | 22.2 | 0.0 | - |
| Hydrogen carbonate buffer | 0.0 | 50.0 | 0.0 | 0.0 | 22.2 | 0.0 | - |
|  |  |  |  |  |  |  |  |
| Triart C18 | 10.0 | 25.0 | 20.0 | 26.7 | 0.0 | 26.7 | - |
| Triart C18 ExRS | 30.0 | 25.0 | 26.7 | 20.0 | 0.0 | 13.3 | - |
| Triart Phenyl | 20.0 | 25.0 | 13.3 | 26.7 | 0.0 | 26.7 | - |
| Acquity BEH C18 | 10.0 | 25.0 | 20.0 | 26.7 | 0.0 | 26.7 | - |
| Luna OMEGA Polar C18 | 30.0 | 0.0 | 20.0 | 0.0 | 100.0 | 6.7 | - |

Table S6 Example of data layout for processing via the R Language script. Retention and overall resolution expressed as peak capacity (PkC):

| ID | COLUMN | ORGANICS | ADDITIVE | pH | RT.Citalo | RT.Trazo | RT.Mirta | RT.Sertra | PkC |
| --- | --- | --- | --- | --- | --- | --- | --- | --- | --- |
| 1 | Triart C18 | Methanol | Formic acid | 2.74 | 4.14 | 3.63 | 2.84 | 5.38 | 38.1 |
| 2 | Triart C18 ExRS | Methanol | Formic acid | 2.74 | 3.96 | 3.45 | 2.68 | 5.36 | 39.9 |
| 3 | Triart C18 | Acetonitrile | Formic acid | 2.74 | 2.95 | 2.51 | 1.76 | 3.59 | 28.5 |
| 4 | Triart C18 ExRS | Acetonitrile | Formic acid | 2.74 | 2.7 | 2.3 | 1.58 | 3.41 | 30.2 |
| 5 | Triart Phenyl | Methanol | Formic acid | 2.74 | 4.44 | 4.27 | 3.26 | 5.55 | 39.6 |
| 6 | Acquity BEH C18 | Methanol | Formic acid | 2.74 | 4.41 | 3.75 | 3.05 | 6.05 | 44.9 |
| 7 | Triart Phenyl | Acetonitrile | Formic acid | 2.74 | 3.02 | 2.67 | 1.85 | 3.66 | 25.5 |
| 8 | Acquity BEH C18 | Acetonitrile | Formic acid | 2.74 | 3.38 | 2.78 | 1.95 | 4.51 | 34.0 |
| 9 | Luna OMEGA Polar C18 | Methanol | Formic acid | 2.74 | 3.95 | 3.49 | 2.75 | 5.16 | 37.2 |
| 10 | Luna OMEGA Polar C18 | Acetonitrile | Formic acid | 2.74 | 2.88 | 2.46 | 1.7 | 3.53 | 26.3 |
| 11 | Triart C18 | Methanol | Acetic acid | 3.34 | 3.86 | 3.43 | 2.96 | 5.04 | 39.4 |
| 12 | Triart C18 ExRS | Methanol | Acetic acid | 3.34 | 3.84 | 3.43 | 2.94 | 5.22 | 40.8 |
| 13 | Triart C18 | Acetonitrile | Acetic acid | 3.34 | 2.62 | 2.27 | 1.83 | 3.18 | 31.7 |
| 14 | Triart C18 ExRS | Acetonitrile | Acetic acid | 3.34 | 2.58 | 2.19 | 1.77 | 3.23 | 32.1 |

Skewness:

| ID | COLUMN | ORGANICS | ADDITIVE | pH | SKEW.Citalo | SKEW.Trazo | SKEW.Mirta | SKEW.Sertra |
| --- | --- | --- | --- | --- | --- | --- | --- | --- |
| 1 | Triart C18 | Methanol | Formic acid | 2.74 | 1.625 | 4.549 | 4.14 | 2.793 |
| 2 | Triart C18 ExRS | Methanol | Formic acid | 2.74 | 2.119 | 2.758 | 4.637 | 4.707 |
| 3 | Triart C18 | Acetonitrile | Formic acid | 2.74 | 0.453 | 1.055 | 2.746 | 5.097 |
| 4 | Triart C18 ExRS | Acetonitrile | Formic acid | 2.74 | 0.941 | 3.014 | 4.889 | 1.86 |
| 5 | Triart Phenyl | Methanol | Formic acid | 2.74 | 0.876 | 2.454 | 1.643 | 1.111 |
| 6 | Acquity BEH C18 | Methanol | Formic acid | 2.74 | 1.399 | 2.686 | 3.517 | 1.915 |
| 7 | Triart Phenyl | Acetonitrile | Formic acid | 2.74 | 0.4 | 2.597 | 2.549 | 0.422 |
| 8 | Acquity BEH C18 | Acetonitrile | Formic acid | 2.74 | 0.791 | 2.335 | 3.754 | 1.377 |
| 9 | Luna OMEGA Polar C18 | Methanol | Formic acid | 2.74 | 2.665 | 3.279 | 5.215 | 2.972 |
| 10 | Luna OMEGA Polar C18 | Acetonitrile | Formic acid | 2.74 | 0.578 | 3.118 | 5.33 | 3.175 |
| 11 | Triart C18 | Methanol | Acetic acid | 3.34 | 1.952 | 2.933 | 5.004 | 5.229 |
| 12 | Triart C18 ExRS | Methanol | Acetic acid | 3.34 | 2.673 | 2.82 | 4.733 | 3.183 |
| 13 | Triart C18 | Acetonitrile | Acetic acid | 3.34 | 0.59 | 2.721 | 3.18 | 1.98 |
| 14 | Triart C18 ExRS | Acetonitrile | Acetic acid | 3.34 | 1.161 | 2.875 | 3.276 | 3.896 |

Table S7 Box-Behnken design optimization of olanzapine and mirtazapine separation. The levels of flow rate and temperature as independent variables (Factor 1 and 2) and corresponding resolution (Response 1).

| **Independent variable** | |  | **Level** | | |
| --- | --- | --- | --- | --- | --- |
|  |  |  | -1 | 0 | +1 |
|  |  |  |  |  |  |
| Flow rate (mL/min) | |  | 0.3 | 0.4 | 0.5 |
| Temperature (°C) | |  | 35.0 | 47.5 | 60.0 |
|  |  |  |  |  |  |
| **Run** | |  | **Factor 1** | **Factor 2** | **Response 1** |
|  |  |  | **Flow rate** | **Temperature** | **Resolution** |
|  |  |  | mL/min | °C | - |
| 1 | |  | 0.3 | 35.0 | 1.43 |
| 2 | |  | 0.3 | 47.5 | 1.33 |
| 3 | |  | 0.3 | 60.0 | 1.93 |
| 4 | |  | 0.4 | 35.0 | 0.74 |
| 5 | |  | 0.4 | 60.0 | 0.75 |
| 6 | |  | 0.5 | 35.0 | 0.58 |
| 7 | |  | 0.5 | 47.5 | 0.33 |
| 8 | |  | 0.5 | 60.0 | 0.98 |
| 9 | |  | 0.4 | 47.5 | 1.53 |

**
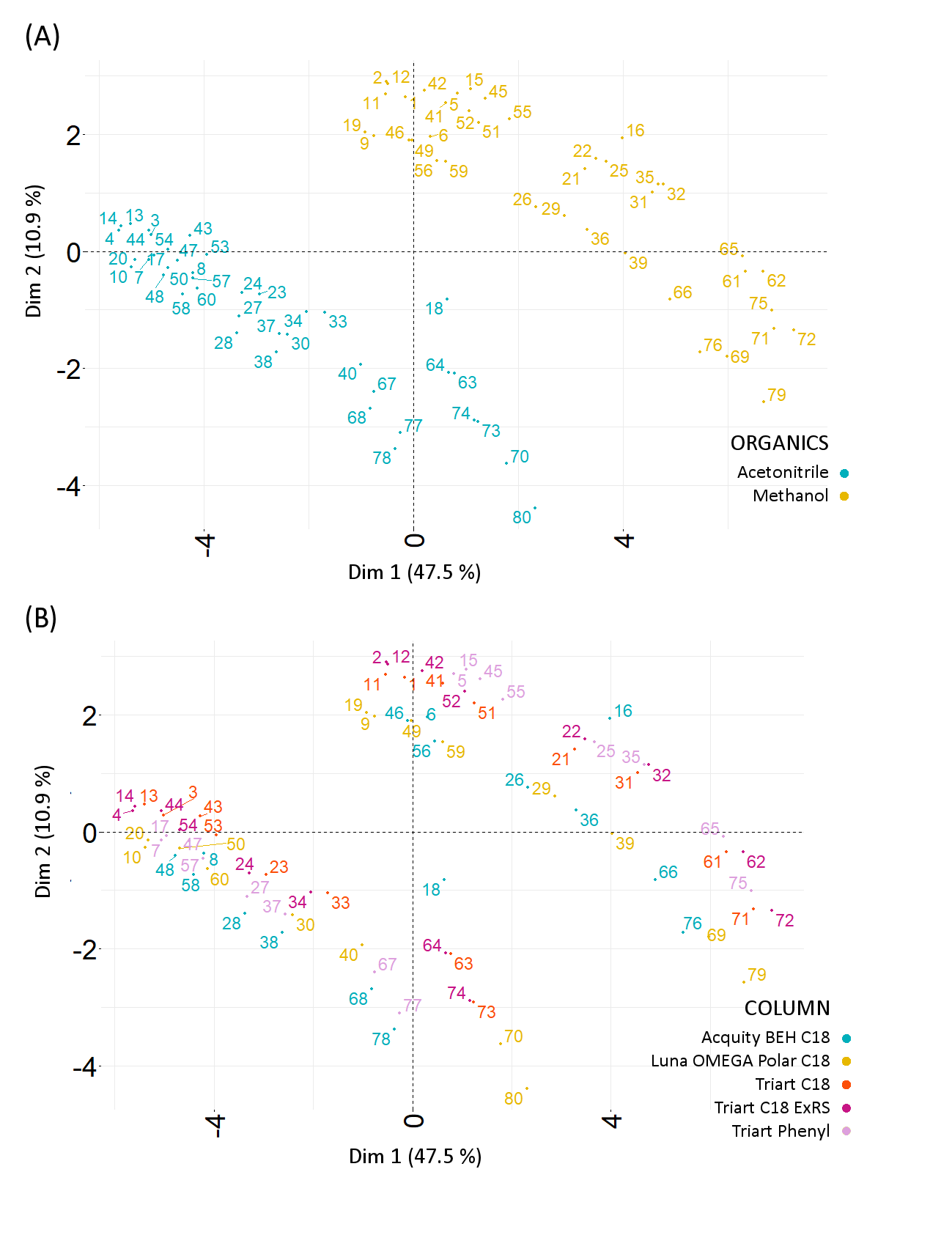
**

Fig. S1 Plot of individuals: Retention investigation. Projection of individuals in principal dimension 1 (Dim 1) and 2 (Dim 2). The numerical annotations correspond to the individual IDs as described in Table S3. Color coding is based on A) Type of organic solvent in the mobile phase, and B) Type of stationary phase used

**
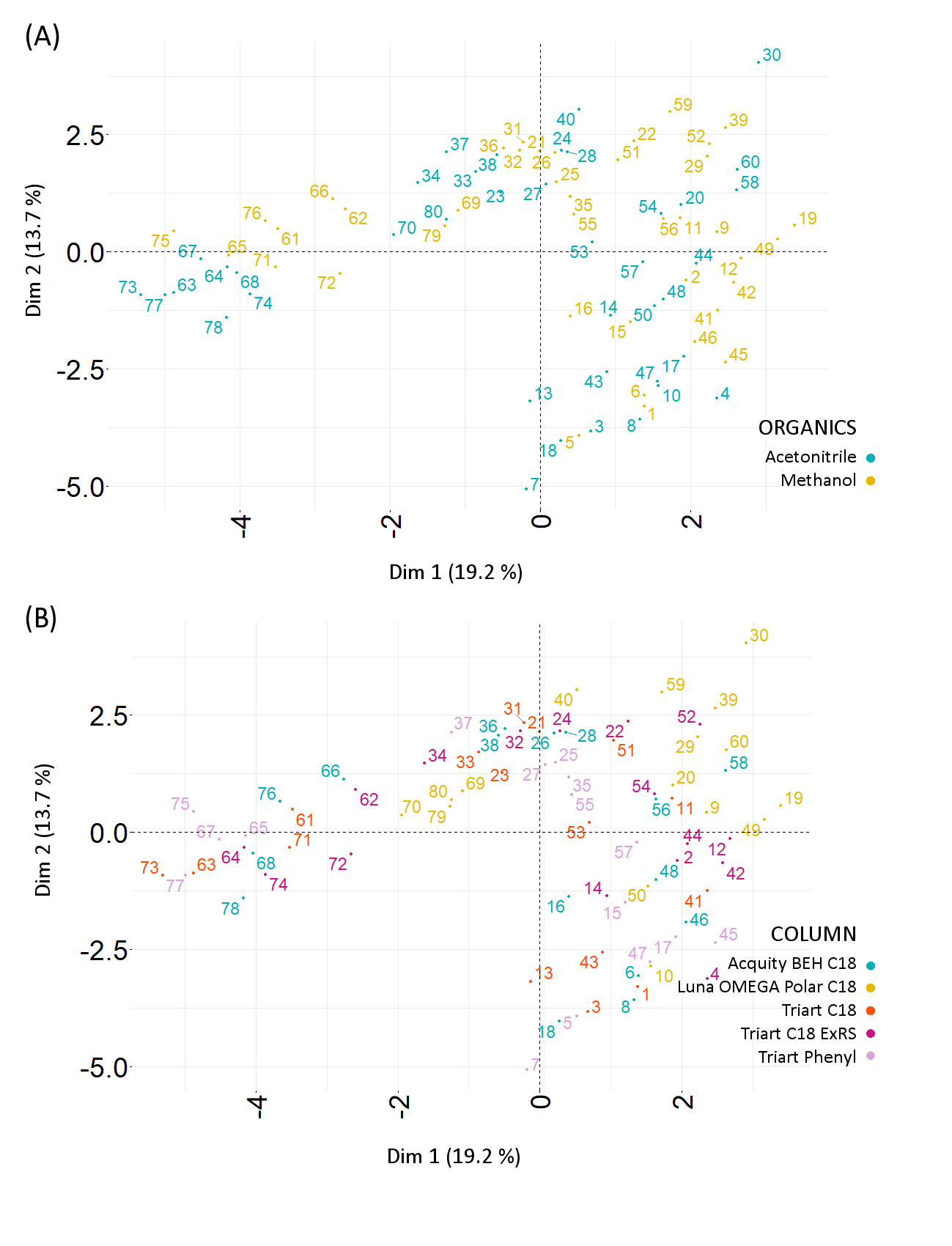
**

Fig. S2 Plot of individuals: Peak skewness investigation. Projection of individuals in principal dimension 1 (Dim 1) and 2 (Dim 2). The numerical annotations correspond to the individual IDs as described in Table S3. Color coding is based on A) Type of organic solvent in the mobile phase, and B) Type of stationary phase used


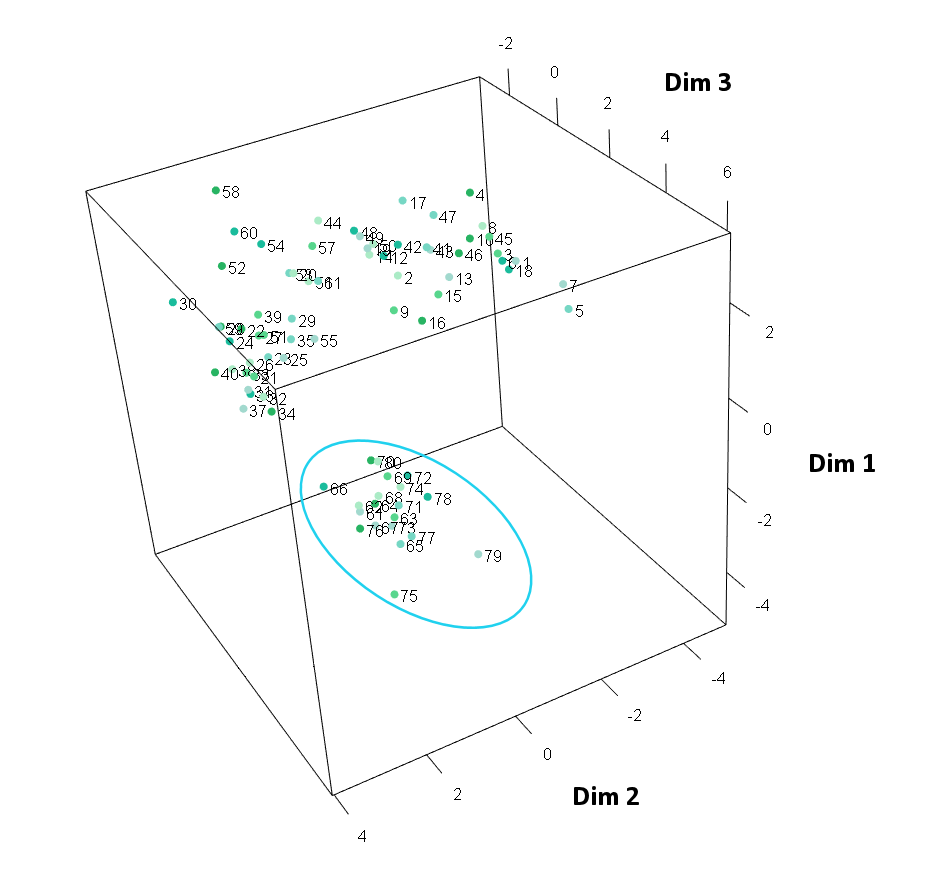


Fig. S3 Three-dimensional plot of individuals: Skewness investigation. Projection of individuals (unique combinations of chromatographic parameters) onto principal dimension 1 (Dim 1), 2 (Dim 2), and 3 (Dim 3), demonstrating the distinct separation of the group using an alkaline additive in the mobile phase (marked with an ellipse)

**
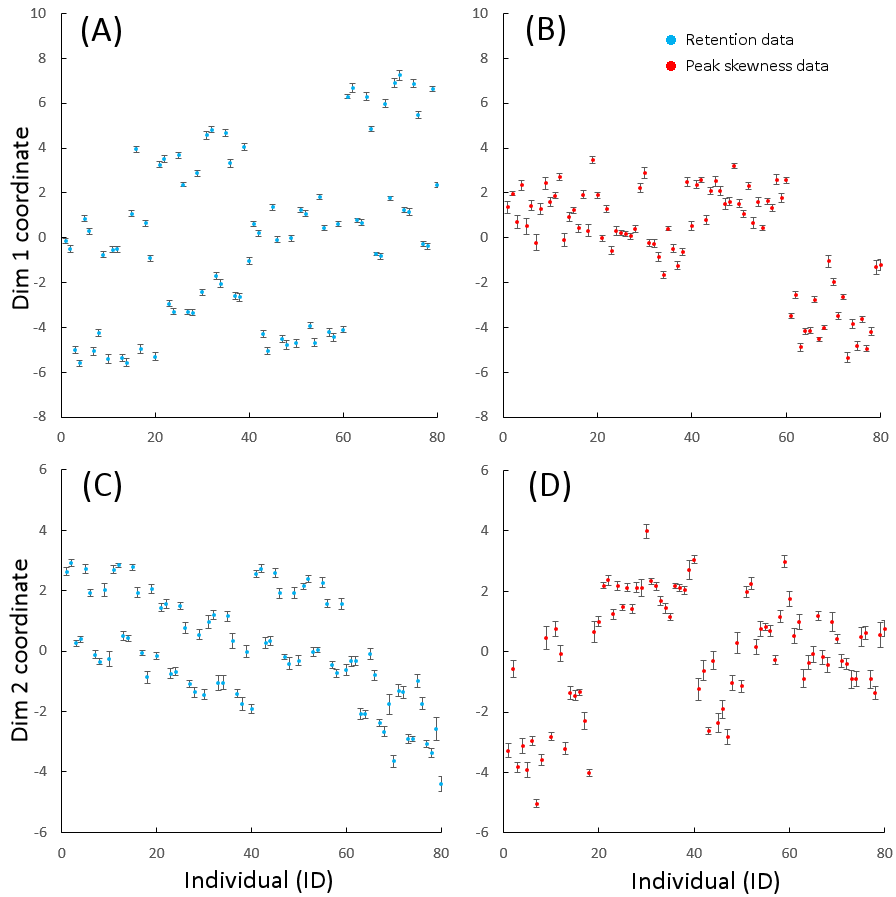
**

Fig. S4 FAMD method K-fold cross-validation (K = 8), showing coordinates stability of individuals in retention and peak skewness evaluation. The coordinates are presented as MEAN ± SD (n = K). Retention (A) Dim 1 and (C) Dim 2 (blue dots). Peak Skewness (B) Dim 1 and (D) Dim 2 (red dots).

**
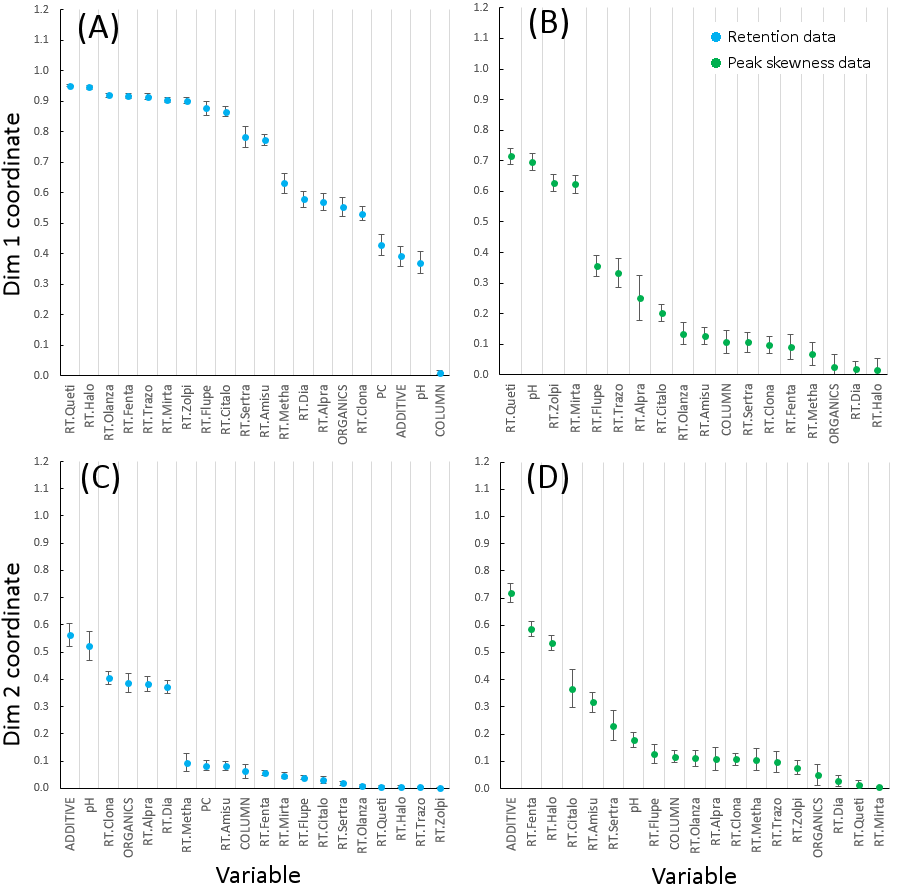
**

Fig. S5 FAMD method K-fold cross-validation (K = 8), showing coordinates stability of variable contributions in retention and peak skewness evaluation. The coordinates are presented as MEAN ± SD (n = 8). Retention (A) Dim 1 and (C) Dim 2 (blue dots). Peak skewness (B) Dim 1 and (D) Dim 2 (green dots).

**
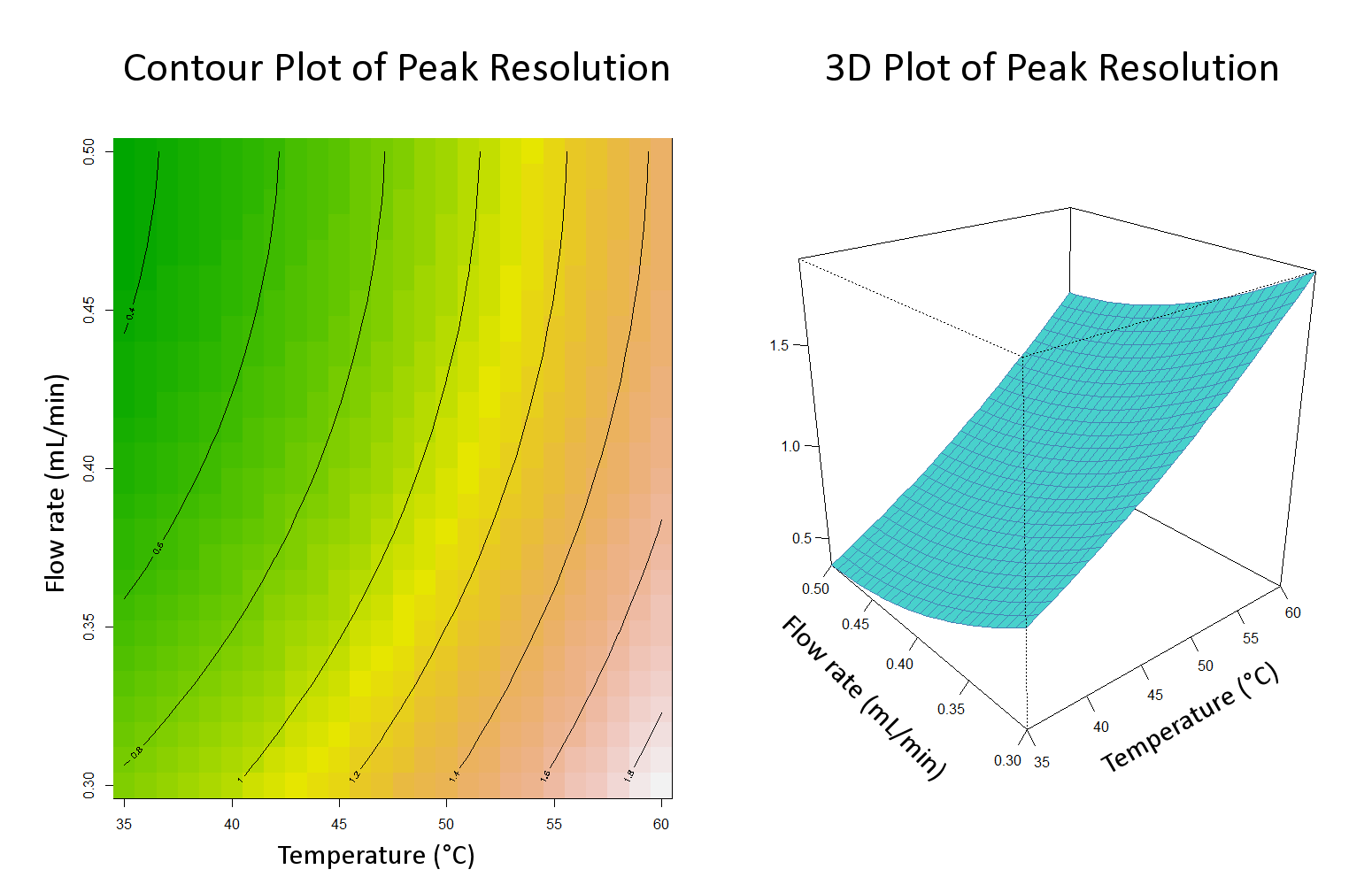
**

Fig. S6 Contour plot and response surface showing dependence of resolution of olanzapine-mirtazapine critical pair on temperature and flow rate as a result of Box-Behnken design optimization

# Preparation of Data for Processing by R Language

### Dataset Structure

The columns in the dataset table contain individual dependent and independent variables. The order of columns is arbitrary. Only a mandatory column is placed at the first place; its header must be 'ID' and contain a unique identification number of the observation (individual). The numbering of IDs starts with digit 1.

The first row of the table contains headers for individual columns. The headers of the rows must be unique and should not be repeated in the dataset. The header string can contain both lowercase and uppercase letters, or their combination. Except for the period, reserved or special characters such as space, comma, brackets, +, -, *, \", /, ~, :, <, >, ;, §, @, #, $, %, ^, &, \", ě, ö, ü, õ, ô, or similar, must not be used in the header string. The header row is followed by rows of individual observations – individuals (combinations of independent chromatographic variables) containing numerical or categorical values, each observation placed on a separate row. There must be no empty row at the end of the dataset or blank cells at the end of the rows! Period has to be used as decimal point delimiter (allowed: 2.458, not allowed: 2,458). Delimiter of orders is not allowed (allowed: 1500800, not allowed: 1 500 800 or 1,500,800). An example of a dataset structure is presented in Table S6.

### Dataset Preparation and Export

Data can be prepared in spreadsheet software such as Microsoft Excel, Open Office Calc or similar and exported as a TAB-delimited text file (*.txt). It is **! VERY IMPORTANT !** to ensure that the data structure does not contain empty cells!!! This means not only missing (N/A) data but also empty rows or columns, which sometimes remain after exporting data from MS Excel to a text file. Using UNICODE text files or other formats is not supported. Failure to adhere to these rules will result in an error, and the calculation will not be performed!

### Dataset for ChromaFAMDeX

All the rules mentioned above apply to the preparation of the dataset for the ChromaFAMDeX application as well.

# R Language Script to Perform FAMD and HC Analysis

The following section presents an R Language script specifically designed for performing Factor Analysis of Mixed Data (FAMD) and Hierarchical Clustering (HC) on datasets derived from optimization experiments in (U)HPLC or LC-MS method development. The script uses the following color coding for better orientation within the code:

- **Steel blue:** The commands of R-language
- Light blue: Important selectors, switches, or user defined parameters
- Brown: User defined variables
- Green: Comments

# -------------------------------------------------------------------------

# **3.1 Installation of necessary libraries**

# -------------------------------------------------------------------------

# Done only once, after fresh installation of R language in a computer

**install.packages**("stats")

**install.packages**("Factoshiny")

**install.packages**("FactoMineR")

**install.packages**("factoextra")

**install.packages**("ggplot2")

**install.packages**("ggrepel")

**install.packages**("rgl")

**install.packages**("ape")

# -------------------------------------------------------------------------

# **3.2 Loading of necessary libraries**

# -------------------------------------------------------------------------

# Necessary to do at each start of the R-studio. Another possibility is to

# include the following code into the Rprofile.site file in the R directory

# C:\Program Files\R\R-4.3.2\etc\

# Library for statistical calculations and analyses

**library**("stats")

# Library for multivariate data analysis

**library**("FactoMineR")

# Extraction and visualization of the output from multivariate data analysis

**library**("factoextra")

# Interactive user interface for performing multivariate data analysis

**library**("Factoshiny")

# Library for visualization of data in 3D plots

**library**("rgl")

# Library for analysis of phylogenetics and evolution used for dendrograms

**library**("ape")

# Versatile environment for data visualizations library

**library**("ggplot2")

# Extension for visualization with the ggplot2 library

**library**("ggrepel")

# -------------------------------------------------------------------------

# **3.3 Loading and formatting data**

# -------------------------------------------------------------------------

# The definition of folders for input data uploading into R-studio

# (input file has to be prepared as a TAB-delimited text file) and saving

# output plots

# Path to the input data file (change the path to your data)

data_path <- "E:/R-project/CNS-drugs/Validated_RT/Raw_data_RT.txt"

# Path where to save the plot outputs (change the path to your output folder)

save_path <- "E:/R-project/CNS-drugs/Validated_RT/"

# Uploading the data file into R-Studio

data_raw <- read.delim(data_path, header = TRUE, sep = "\t", dec = ".")

# Visualization of the first six rows of the datafile

**head**(data_raw)

# Extraction of the data, while skipping the first column containing ID number

dataset <- data_raw[,2:ncol(data_raw)]

**head**(dataset)

# Extraction of the ID numbers from original data file

row_ID <- data_raw[,1]

**head**(row_ID)

# -------------------------------------------------------------------------

# **3.4 FAMD analysis**

# Customized code for complete FAMD analysis performed on chromatographic data

# -------------------------------------------------------------------------

# Initial FAMD Analysis

# Performing the FAMD analysis on the dataset. The result is stored in

# ‘res.famd’ object

res.famd <- **FAMD**(dataset, graph = FALSE)

# Printing a summary of the FAMD analysis results

**print**(res.famd)

# Extracting the eigenvalues from the result of the FAMD analysis.

# Eigenvalues indicate the amount of variance captured by each principal dimension

eig.val <- **get_eigenvalue**(res.famd)

# Visualization of the first six eigenvalues

**head**(eig.val)

# Performing a descriptive analysis of the first two principal dimensions

# c(1,2), identifying variables significantly associated with each principal dimension

# at a 5% significance level (proba = 0.05)

res.desc <- **dimdesc**(res.famd, axes = c(1,2), proba = 0.05)

# Accessing descriptions of principal dimension 1 and 2

res.desc$Dim.1

res.desc$Dim.2

# Variables Analysis

# Extracting and displaying variables from the FAMD output object (res.famd)

var <- **get_famd_var**(res.famd)

var

# Displaying coordinates of the first six variables

**head**(var$coord)

# Displaying quality of representation on the factorial map (1^st^ sixt values)

**head**(var$cos2)

# Displaying contributions to the principal dimensions (1^st^ sixt values)

**head**(var$contrib)

# Visualization

# Extracting and displaying the information on the quantitative variables

quanti.var <- **get_famd_var**(res.famd, "quanti.var")

quanti.var

# Extracting detailed information regarding the qualitative variables

quali.var <- **get_famd_var**(res.famd, "quali.var")

quali.var

# Extracting detailed information regarding the individuals

ind <- **get_famd_ind**(res.famd)

ind

# Scree plot to visualize the eigenvalues

Screeplot <- **fviz_screeplot**(res.famd, addlabels = TRUE, labelsize = 50, repel = TRUE, barfill = "#41b0b2")

# Plot of Variability contribution to Dim 1

Contribution_dim1 <- **fviz_contrib**(res.famd, "var", axes = 1, fill = "steelblue", labelsize = 8)

# Plot of Variability contribution to Dim 2

Contribution_dim2 <- **fviz_contrib**(res.famd, "var", axes = 2, fill = "steelblue", labelsize = 8)

# Correlation circle to visualize relationships between quantitative (numerical) variables and

# principal dimensions

Correlation_Cycle <- **fviz_famd_var**(res.famd, "quanti.var", col.var = "contrib", gradient.cols = **c**("#00AFBB", "#E7B800", "#FC4E07"), labelsize = 12, repel = TRUE)

# Plot of individuals colored according to stationary phase, highlighting potential groupings or

# patterns

Individuals_column <- **fviz_mfa_ind**(res.famd, habillage = "COLUMN", palette = c("#00AFBB", "#E7B800", "#FC4E07", "#C71585", "#DDA0DD", "#00BFFF"), addEllipses = FALSE, ellipse.type = "confidence", mean.point = FALSE, labelsize = 8, repel = TRUE)

# Plot of individuals colored according to mobile phase additive, highlighting potential groupings

# or patterns

Individuals_additive <- **fviz_mfa_ind**(res.famd, habillage = "ADDITIVE", palette = c("#9ACD32", "#FF7F50", "#FF69B4", "#FFD700", "#FF1493", "#FF0000","#4682B4", "#7B68EE", "#20B2AA"), addEllipses = FALSE, ellipse.type = "confidence", mean.point = FALSE, labelsize = 8, repel = TRUE)

# Plot of individuals colored according to mobile phase organic modifier, highlighting potential

# groupings or patterns

Individuals_organics <- **fviz_mfa_ind**(res.famd, habillage = "ORGANICS", palette = c("#00afbb", "#e7b800"), addEllipses = FALSE, ellipse.type = "norm", ellipse.level = 0.95, mean.point = FALSE, labelsize = 8, repel = TRUE)

# Factorial map of qualitative variables to visualize relationships between variables and

# principal dimensions

Factorial_map <- **fviz_famd_var**(res.famd, "quali.var", col.var = "contrib", gradient.cols = **c**("#00AFBB", "#E7B800", "#FC4E07"), labelsize = 8, repel = TRUE)

# Plot of variables contribution to the principal dimensions identified in FAMD

Variables <- **fviz_famd_var**(res.famd, col.var = "contrib", gradient.cols = **c**("#00AFBB", "#E7B800", "#FC4E07"), labelsize = 8, repel = TRUE)

# Common plot of individuals and factors

Individuals_Factors <- **fviz_famd_ind**(res.famd, axes = c(1,2), geom = c("point", "text"), shape.ind = 19, habillage = "ADDITIVE", palette = c("#9ACD32", "#FF7F50", "#FF69B4", "#FFD700", "#FF1493", "#FF0000","#4682B4", "#7B68EE", "#20B2AA"), labelsize = 8, repel = TRUE)

# ------------------------ 3D plot of individuals -----------------------

# The following code generates an interactive 3D plot in a separate window.

# The code is useful for inspection of individuals distribution in 3D space

# if there is considerable variability along the third principal dimension.

# Extracting the first three coordinates for each individual from the ind$coord object

data<- ind$coord[,1:3]

# Defining a vector of colors to be used in 3D plotting

colors = **c**("#a2d9ce", "#ABEBC6", "#58D68D", "#28B463", "#76D7C4", "#1ABC9C")

# Plotting the first three principal dimensions of the data in a 3D scatter plot

**plot3d**(data, size=8, col=**rep**(colors, length.out = **nrow**(data)), main = "*Place your caption here*")

# Add text labels to each point in the 3D plot

**text3d**(data, texts=row_ID, cex=1, pos=4)

# --------------------------------------------------------------------------

# **3.5 Hierarchical clustering**

# --------------------------------------------------------------------------

# The code for the calculation and visualization of hierarchical clusters

# using circular dendrogram. The distance matrix is calculated for a dataset

# df.hc based on FAMD coordinates of individuals

# Extracting coordinates of individuals from FAMD

df.hc <- res.famd$ind$coord

# Calculating distance matrix using the Euclidean distance method

res.dist <- **get_dist**(df.hc, method = "euclidean")

# Calculating hierarchical clusters using Ward’s method

res.hc <- **hclust**(res.dist, method = "ward.D2")

# Defining vector of colors for cluster coloring

colors = **c**("#FFD700", "#FF1493", "blue","#4682B4", "#7B68EE", "#20B2AA","#00afbb")

# Coloring of the dendrogram according to arbitrary division into 6 clusters

clus = **cutree**(res.hc, 6)

# -----------------------------------------------------------------------------

# **3.6 The plots output to R-studio environment**

# -----------------------------------------------------------------------------

# Displaying graphs in the R-Studio 'PLOTS' panel

Screeplot

Contribution_dim1

Contribution_dim2

Correlation_Cycle

Individuals_additive

Individuals_column

Individuals_organics

Factorial_map

Individuals_Factors

Variables

# --------------------------------------------------------------------------

# **3.7 The plots formatting prior to printing**

# --------------------------------------------------------------------------

# Customization of the plots intended for saving on a hard drive (title and label colors, sizes,

# adjustments, etc.)

# Adding labels and captions to the plots

my_labs <- **labs**(title = "*Place your title here* ", subtitle = "*Place your subtitle here* ", caption = "*Place your caption here* ")

# Labels and axes formatting

my_theme <- **theme**(axis.text.x = element_text(

face = "plain",

color = "black",

size = 28,

angle = 90,

vjust = 0.5,

hjust = 1

),

axis.text.y = element_text(

face = "plain",

color = "black",

size = 28,

angle = 0

),

axis.title.x = element_text(size = 40, color = "black"),

axis.title.y = element_text(size = 40, color = "black"),

legend.position = "right",

plot.title = element_text(color = "black", size = 22, face = "bold"),

plot.subtitle = element_text(color = "black", size = 16),

plot.caption = element_text(color = "black", size = 16, face = "italic") )

# Application of the formatting on the plots

Screeplot <- Screeplot + my_theme + my_labs

Contribution_dim1 <- Contribution_dim1 + my_theme + my_labs

Contribution_dim2 <- Contribution_dim2 + my_theme + my_labs

Correlation_Cycle <- Correlation_Cycle + my_theme + my_labs

Individuals_additive <- Individuals_additive + my_theme + my_labs

Individuals_column <- Individuals_column + my_theme + my_labs

Individuals_organics <- Individuals_organics + my_theme + my_labs

Factorial_map <- Factorial_map + my_theme + my_labs

Individuals_Factors <- Individuals_Factors + my_theme + my_labs

Variables <- Variables + my_theme + my_labs

# -----------------------------------------------------------------------------

# **3.8 Printing and saving of the plots**

# -----------------------------------------------------------------------------

# Specifying the print size, file name, and directory for the plots saving

Print_size_X <- 1500 # Width of the image in pixels

Print_size_Y <- 1000 # Height of the image in pixels

my_path <- **paste0**(save_path, "/", "Screeplot.tiff")

**tiff**((file = my_path), width = Print_size_X, height = Print_size_Y)

**print**(Screeplot)

**dev.off**()

my_path <- **paste0**(save_path, "/", " Contribution_dim1.tiff")

**tiff**((file = my_path), width = Print_size_X, height = Print_size_Y)

**print**(Contribution_dim1)

**dev.off**()

my_path <- **paste0**(save_path, "/", " Contribution_dim2.tiff")

**tiff**((file = my_path), width = Print_size_X, height = Print_size_Y)

**print**(Contribution_dim2)

**dev.off**()

my_path <- **paste0**(save_path, "/", "Correlation-Cycle.tiff")

**tiff**((file = my_path), width = Print_size_X, height = Print_size_X)

**print**(Correlation_Cycle)

**dev.off**()

my_path <- **paste0**(save_path, "/", "Individuals-column.tiff")

**tiff**((file = my_path), width = Print_size_X, height = Print_size_Y)

**print**(Individuals_column)

**dev.off**()

my_path <- **paste0**(save_path, "/", "Individuals-additive.tiff")

**tiff**((file = my_path), width = Print_size_X, height = Print_size_Y)

**print**(Individuals_additive)

**dev.off**()

my_path <- **paste0**(save_path, "/", "Individuals-organics.tiff")

**tiff**((file = my_path), width = Print_size_X, height = Print_size_Y)

**print**(Individuals_organics)

**dev.off**()

my_path <- **paste0**(save_path, "/", "Factorial-map.tiff")

**tiff**((file = my_path), width = Print_size_X, height = Print_size_Y)

**print**(Factorial_map)

**dev.off**()

my_path <- **paste0**(save_path, "/", "Individuals-Factors.tiff")

**tiff**((file = my_path), width = Print_size_X, height = Print_size_Y)

**print**(Individuals_Factors)

**dev.off**()

my_path <- **paste0**(save_path, "/", "Variables.tiff")

**tiff**((file = my_path), width = Print_size_X, height = Print_size_Y)

**print**(Variables)

**dev.off**()

lbl_size <- 3.0 # Size of the labels in the dendrogram

lbl_offset <- 0.2 # Offset of the labels in the dendrogram

my_path <- **paste0**(save_path, "/", "Cluster-plot-ward-D2.tiff")

**tiff**((file = my_path), width = Print_size_X, height = Print_size_X)

**plot**(**as.phylo**(res.hc), type = "fan", tip.color = colors[clus], label.offset = lbl_offset, cex = lbl_size, main = "ward.D2")

**dev.off**()

# R Language Script to Perform Normality Test and Spearman’s Correlation Analysis

# -------------------------------------------------------------------------

# **5.1** **Installation of the necessary libraries**

# -------------------------------------------------------------------------

# Done only once, after fresh installation of R language in a computer

**install.packages**("PerformanceAnalytics")

**install.packages**("Hmisc")

# -------------------------------------------------------------------------

# **5.2 Loading of necessary libraries**

# -------------------------------------------------------------------------

# Necessary to do at each start of the R-studio. Another possibility is to

# include the following code into the Rprofile.site file in the R directory

# C:\Program Files\R\R-4.3.2\etc\

**library**("PerformanceAnalytics")

**library**("Hmisc")

# --------------------------------------------------------------------------

# **5.3 Data import**

# --------------------------------------------------------------------------

# Path to the input data file containing NUMERIC VARIABLES ONLY !!!

# (change the path to your data)

data_path <- "E:/R-project/CNS-drugs/_SET33/SET33.txt"

# Path where to save the plot outputs (change the path to your output folder)

save_path <- "E:/R-project/CNS-drugs/_SET33/"

# Uploading the data file to R-Studio. The data file must contain only

# columns with numerical variables

data_raw <- **read.delim**(data_path, header = TRUE, sep = "\t", dec = ".")

# Visualization of the first six rows of the datafile

**head**(data_raw)

# Data extracting except the first column containing ID number

dataset <- data_raw[,2:**ncol**(data_raw)]

**head**(dataset)

# --------------------------------------------------------------------------

# **5.4 Shapiro–Wilk test**

# --------------------------------------------------------------------------

# Normality testing

results <- **apply**(dataset, 2, **function**(column) {**shapiro.test**(column)})

# Command to extract the P-values from the list of test results:

p_values <- **sapply**(results, **function**(x) x$p.value)

# Print the p-values for each column

**print**(p_values)

# Saving the p-values to a text file

normality_results <- **data.frame**(Column = names(p_values), P_Value = p_values)

file_path <- **paste0**(save_path, "/", "normality-test.txt")

**write.table**(normality_results, file = file_path, sep = "\t", row.names = FALSE, quote = FALSE)

# --------------------------------------------------------------------------

# **5.5 Spearman’s correlation coefficient**

# --------------------------------------------------------------------------

# Calculating Spearman's rank correlation for nonparametric data

spearman_correlation_matrix <- **cor**(dataset, method = "spearman")

# Showing the correlation matrix in the R-Studio

**print**(spearman_correlation_matrix)

# Saving Spearman’s correlation coefficients to a text file on a hard drive

file_path <- **paste0**(save_path, "/", "spearmans_corr.txt")

**write.table**(spearman_correlation_matrix, file = file_path, sep = "\t", row.names = FALSE, quote = FALSE)

# Saving plot of the correlation matrix to a hard drive

my_path <- **paste0**(save_path, "/", "corr_matrix.tiff")

**tiff**((file = my_path), width = 2500, height = 2500)

**chart.Correlation**(dataset, method = "spearman", histogram=TRUE, pch=19)

**dev.off**()

# Calculating Spearman's rank correlations and p-values

cor_and_pvalues <- **rcorr**(**as.matrix**(dataset), type = "spearman")

# Extracting the correlation matrix

spearman_correlation_matrix <- cor_and_pvalues$r

# Extracting the matrix of P-values

p_values_matrix <- cor_and_pvalues$P

# Printing the correlation matrix in the R-Studio

**print**(spearman_correlation_matrix)

# Printing the p-values matrix in R-Studio

**print**(p_values_matrix)

# Defining the file paths

corr_path <- **paste0**(save_path, "/", "spearmans_corr.txt")

p_path <- **paste0**(save_path, "/", "spearmans_pvalues.txt")

# Save the correlation matrix to hard drive in a text file

**write.table**(spearman_correlation_matrix, file = corr_path, sep = "\t", row.names = FALSE, quote = FALSE)

# Save the p-values matrix to the hard drive in a text file

**write.table**(p_values_matrix, file = p_path, sep = "\t", row.names = FALSE, quote = FALSE)

# R Language Script to Perform K-fold cross-validation

The following section presents an R Language script intended for performing K-fold cross-validation of FAMD analysis.

# -------------------------------------------------------------------------

# **4.1** **Installation of the necessary libraries**

# -------------------------------------------------------------------------

# Done only once, after fresh installation of R language in a computer

**install.packages**("caret")

**install.packages**("ggplot2")

**install.packages**("ggrepel")

**install.packages**("dplyr")

# -------------------------------------------------------------------------

# **4.2 Loading of necessary libraries**

# -------------------------------------------------------------------------

# Necessary to do at each start of the R-studio. Another possibility is to

# include the following code into the Rprofile.site file in the R directory

# C:\Program Files\R\R-4.3.2\etc\

**library**("caret")

**library** ("ggplot2")

**library** ("ggrepel")

**library** ("dplyr")

# -------------------------------------------------------------------------

# **4.3 Loading and formatting data**

# -------------------------------------------------------------------------

# Path to the input data file (change the path to your data)

data_path <- "E:/R-project/CNS-drugs/Validated_RT/Raw_data_RT.txt"

# Path where to save the outputs (change the path to your preferred folder)

save_path <- "E:/R-project/CNS-drugs/Validated_RT/"

Result_KFold_coord <- **paste0**(save_path, "KFold_coord_RT.txt")

Result_KFold_mean_sd <- **paste0**(save_path,"KFold_mean_sd_RT.txt")

# Uploading the data file into R-Studio

data_raw <- read.delim(data_path, header = TRUE, sep = "\t", dec = ".")

# Extraction of the data, while skipping the first column containing only ID number

dataset <- data_raw[,2:**ncol**(data_raw)]

# Extraction of the ID numbers from original data file

row_ID <- data_raw[,1]

# -------------------------------------------------------------------------

# **4.4 K-fold cross-validation**

# -------------------------------------------------------------------------

# Define the number of folds

fold_number <- 8

# Create folds for cross-validation

folds <- **createFolds**(dataset[[1]], k = fold_number)

# Function to train FAMD

trainFAMD <- **function**(train_data) {

famd_res <- **FAMD**(train_data, ncp = 5, graph = FALSE)

**return**(famd_res)

}

# Perform K-Fold cross-validation

results <- **lapply**(seq_along(folds), function(i) {

fold_indices <- folds[[i]]

train_data <- dataset[-fold_indices, ]

test_data <- dataset[fold_indices, ]

famd_model <- trainFAMD(train_data)

# Get row IDs for this fold

row_ID <- **rownames**(train_data)

**return**(**list**(fold = i, model = famd_model))

})

# Function to save results to a text file

saveResults <- **function**(results) {

all_coords <- **do.call**(**rbind**, **lapply**(results, **function**(res) {

ind_coords <- **as.data.frame**(res$model$ind$coord)

ind_coords$ID <- **rownames**(ind_coords)

**return**(ind_coords)

}))

**write.table**(all_coords, file = Result_KFold_coord, sep = "\t", row.names = FALSE, col.names = TRUE)

}

# Save the results

**saveResults**(results)

# -------------------------------------------------------------------------

# **4.5 Calculation of mean and SD after cross-validation**

# -------------------------------------------------------------------------

# Read the data from the text file

data <- **read.table**(Result_KFold_coord, header = TRUE)

# Calculate mean and standard deviation for coordinates obtained from K-fold method

summary_data <- data %>%

**group_by**(ID) %>%

**summarise**(

mean_value_Dim.1 = **mean**(Dim.1, na.rm = TRUE),

sd_value_Dim.1 = **sd**(Dim.1, na.rm = TRUE),

mean_value_Dim.2 = **mean**(Dim.2, na.rm = TRUE),

sd_value_Dim.2 = **sd**(Dim.2, na.rm = TRUE),

)

# Save the result

**write.table**(summary_data, file = Result_KFold_mean_sd, row.names = FALSE, quote = FALSE)

# Box-Behnken design optimization

The following section presents an R Language script designed for planning of Box-Behnken design optimization and evaluation.

# -------------------------------------------------------------------------

# **6.1** **Installation of the necessary libraries**

# -------------------------------------------------------------------------

# Done only once, after fresh installation of R language in a computer

**install.packages**("rsm")

# -------------------------------------------------------------------------

# **6.2 Loading of necessary libraries**

# -------------------------------------------------------------------------

# Necessary to do at each start of the R-studio. Another possibility is to

# include the following code into the Rprofile.site file in the R directory

# C:\Program Files\R\R-4.3.2\etc\

**library**("rsm")

# -------------------------------------------------------------------------

# **6.2 Generating the Box-Behnken design**

# -------------------------------------------------------------------------

# Path where to save the design

save_path <- "E:/R-project/CNS-drugs/Validated_RT/BBD_design.txt"

# Definition of the extreme levels (min/max) for the factors

temperature_range <- c(35, 60)

flow_rate_range <- c(0.3, 0.5)

# Generating coded levels

coded_design <- **expand.grid**(temp = c(-1, 0, 1), flow = c(-1, 0, 1))

coded_design <- coded_design[!(coded_design$temp == 0 & coded_design$flow == 0), ]

coded_design <- **rbind**(coded_design, c(0, 0))

# Converting the coded levels to actual levels

actual_design <- coded_design

actual_design$temp <- (coded_design$temp + 1) / 2 * (temperature_range[2] - temperature_range[1]) + temperature_range[1]

actual_design$flow <- (coded_design$flow + 1) / 2 * (flow_rate_range[2] - flow_rate_range[1]) + flow_rate_range[1]

# Print the actual design

**print**(actual_design)

# Save design in a text file

**write.table**(actual_design, file = save_path, sep = "\t", row.names = FALSE, quote = FALSE)

# --------------------------------------------------------------------------------

# **6.2 Evaluation of Box-Behnken design-based optimization**

# --------------------------------------------------------------------------------

# Path to the input data file (change the path to your data)

data_path <- "E:/R-project/CNS-drugs/Validated_RT/BBD_input.txt"

# Uploading the data file into R-Studio

dataset <- **read.delim**(data_path, header = TRUE, sep = "\t", dec = ".")

# The columns in the input text file are named as follows: Flowrate, Temperature, Resolution

# If the column names are different, adjust accordingly

**colnames**(dataset) <- c("Flowrate", "Temperature", "Resolution")

# Print the data to verify

**print**(dataset)

# Fit the response surface model using second-order polynomial

model <- **rsm**(Resolution ~ SO(Temperature, Flowrate), data = dataset)

# Print the summary of the model

**summary**(model)

**par**(**mfrow** = **c**(1, 2))

**contour**(model, ~ Temperature + Flowrate,

image = TRUE,

main = "Contour Plot of Peak Resolution")

**persp**(model, ~ Temperature + Flowrate,

theta = 320, phi = 25,

border = "Steel Blue",

col ="MediumTurquoise",

main = "3D Plot of Peak Resolution",

)
